# *TaWAK6* encoding wall-associated kinase is involved in wheat resistance to leaf rust similar to adult plant resistance

**DOI:** 10.1371/journal.pone.0227713

**Published:** 2020-01-13

**Authors:** Marta Dmochowska-Boguta, Yuliya Kloc, Andrzej Zielezinski, Przemysław Werecki, Anna Nadolska-Orczyk, Wojciech M. Karlowski, Wacław Orczyk

**Affiliations:** 1 Department of Genetic Engineering, Plant Breeding and Acclimatization Institute-National Research Institute, Radzikow, Blonie, Poland; 2 Department of Computational Biology, Institute of Molecular Biology and Biotechnology, Faculty of Biology, Adam Mickiewicz University, Poznan, Poland; 3 Department of Functional Genomics, Plant Breeding and Acclimatization Institute-National Research Institute, Radzikow, Blonie, Poland; Institute of Genetics and Developmental Biology Chinese Academy of Sciences, CHINA

## Abstract

In wheat, adult plant resistance (APR) to leaf rust (*Puccinia triticina*), is effective in restricting pathogen growth and provides durable resistance against a wide range of virulent forms of *P*. *triticina*. Despite the importance, there is limited knowledge on the molecular basis of this type of resistance. We isolated and characterized the wall-associated kinase encoding gene in wheat, and assigned it as *TaWAK6*. Localization of *TaWAK6* homeologs in A and B wheat subgenomes was consistent with the presence of the gene’s orthologs in *T*. *urartu* (AA) and *T*. *dicoccoides* (AABB) and with the absence of its orthologs in *Aegilops tauschii* (DD). Overexpression of *TaWAK6* did not change the wheat phenotype, nor did it affect seedling resistance. However, the adult plants overexpressing *TaWAK6* showed that important parameters of APR were significantly elevated. Infection types scored on the first (flag), second and third leaves indicated elevated resistance, which significantly correlated with expression of *TaWAK6*. Analysis of plant-pathogen interactions showed a lower number of uredinia and higher rates of necrosis at the infection sites and this was associated with smaller size of uredinia and a longer latent period. The results indicated a role of *TaWAK6* in quantitative partial resistance similar to APR in wheat. It is proposed that TaWAK6, which is a non-arginine-aspartate (non-RD) kinase, represents a novel class of quantitative immune receptors in monocots.

## Introduction

Wall-associated kinases (WAKs) are a class of receptor protein kinases localized in the cell wall with the function of sensing environmental and cellular signals. They have the structure of receptor-like proteins and contain an extracellular, a transmembrane and intracellular domains. The extracellular domain is built of several epidermal growth factor (EGF) repeats and, in association with the transmembrane domain, forms bonds with the cell wall [1]. The cytoplasmic domain of serine threonine kinase has a function in activating signaling cascades [2]. WAKs can bind pectin polymers or pectin fragments released as a result of cell development, mechanical damage or pathogen infection [3]. This in turn triggers signaling, which activates different types of responses [4, 5]. Binding of native pectins acts as signals of cell expansion, while binding of oligogalacturonides (OGs) activates stress signaling [3, 4]. In response to biotic stresses the OGs participate in production of nitric oxide (NO) and reactive oxygen species (ROS) [6] and, in association with WAKs, function as damage-associated molecular patterns (DAMPs) triggering plant innate immunity [7]. There is growing evidence that WAKs play important roles in host basal resistance. Diener and Ausubel [8] for the first time reported that wall-associated kinase (namely WAKL22) was involved in the dominant and race non-specific plant resistance. The maize *qHSR1* locus conferring quantitative resistance against *Sporisorium reilianum* causing head smut disease was found to encode a protein with all the features of wall-associated kinase [9]. A further study by Zhang *et al*. [10] revealed that *ZmWAK* functioned as a molecular switch promoting growth in the absence of *S*. *reilianum* and activating apoptosis-like defense upon pathogen infection. Maize resistance against *Exserohilum turcicum* causing northern corn leaf blight, conferred by the *Htn1* locus, was found to contain 3 candidate genes all encoding wall-associated kinases. One of them, *ZmWAK-RLK1*, was found to confer the resistant phenotype. The non-arginine-aspartate (non-RD) kinase domain of the protein, encoded by *ZmWAK-RLK1*, represented a novel class of quantitative immune receptors in monocots [11]. In rice the *OsWAK1* gene was found to be significantly induced during incompatible interaction with the rice blast fungus *Magnaporthe oryzae*, after mechanical wounding and salicylic acid or methyl jasmonate treatment. Overexpression of the gene in rice enhanced the resistance against rice blast [12]. Delteil *et al*. [13] analyzed 4 OsWAK proteins and found that they functioned as regulators of rice quantitative resistance against the rice blast fungus *M*. *oryzae*. Three of the tested WAKs (*OsWAK14*, *OsWAK91* and *OsWAK92*) positively regulated quantitative resistance while *OsWAK112d* functioned as a negative regulator [13, 14]. Overexpression of another rice gene, *OsWAK25*, caused lesion mimic phenotype and activated expression of rice pathogenesis-related genes *PR10*, *OsPAL2*, *PBZ1* and *NH1*. The *OsWAK25* overexpressing plants were more resistant to the hemibiotrophic pathogens *Xanthomonas oryzae* and *M*. *oryzae*, and at the same time more susceptible to the necrotrophic pathogens *Rhizoctonia solani* and *Cochliobolus miyabean* [15]. Recently Hu, Cao [16] reported that the rice *Xa4* locus, known to confer durable resistance against *Xanthomonas oryzae* pv. *oryzae*, encoded wall-associated kinase. The resistance conferred by *Xa4* was associated with promoting cellulose synthesis, suppressing cell wall loosening and overall strengthening of the cell wall. Tomato wall-associated kinase *SlWAK1* was identified as one of the *FIRE* (*Flagellin-induced repressed by effectors*) genes in tomato [17]. The recognition of microbe associated molecular patterns by plant receptors triggered *SlWAK1* expression, which in turn activated a sustained immune response. Virus-induced silencing of the gene compromised plant resistance [17].

Wheat resistance against biotic factors is crucial to food security. The crop is one of the most important food producers, second only to rice. There is growing evidence that wall-associated kinases play important functions in the wheat immune system. The *Stb6* locus in wheat confers field resistance against septoria tritici blotch (STB) disease caused by *Zymoseptoria tritici*. The locus found in archeological wheat accessions is believed to have provided durable resistance against STB since the mid-Neolithic period [18]. Map-based cloning of the *Stb6* locus identified a region of wheat chromosome 3A with 6 wall-associated kinase-like *TaWAKL* genes. The detailed functional analysis revealed that one of them, namely *TaWAKL4*, was responsible for the resistance against *Z*. *tritici*. It is worth noting that the *Stb6*-based plant resistance does not involve the hypersensitive response and in this respect it is similar to the broad range type of resistance [19]. Another wheat wall-associated receptor kinase, W5G2U8_WHEAT, has been found to function as a receptor of chitosan oligosaccharides, which function as molecules activating the plant immune system and enhancing resistance to pests and diseases [20]. The *TaWAK5* gene was isolated based on expression profiles differentiating wheat lines resistant and susceptible to eyespot disease caused by *Rhizoctonia cerealis*. The gene was also induced by exogenous salicylic acid, abscisic acid, and methyl jasmonate. The TaWAK5 protein, localized to the plasma membrane, contains a signal peptide, epidermal growth factor (EGF)-like repeats, a transmembrane domain and a serine/threonine protein kinase catalytic domain. Silencing of the gene in the resistant genotype did not impair wheat resistance against *R*. *cerealis*. According to the authors, the results implied that the gene may be functionally redundant with other genes [21]. The *Snn1* locus conferring wheat susceptibility to the necrotrophic pathogen *Parastagonospora nodorum* was found to contain a WAK-encoding gene. Interaction of the kinase with the pathogen’s virulence factor SnTox1 activated programmed cell death and promoted pathogen growth. The authors demonstrated that the WAK protein typically involved in resistance against biotrophic pathogens was, in this particular case, hijacked by a necrotrophic pathogen to colonize the plant cells [22].

Currently 79 leaf rust resistance genes (*Lr*) are known. Most of them confer all-stage resistance (ASR). ASR is often race-specific and, due to pathogen evolution and novel virulences, it could become ineffective within a few years after introduction. Fifteen *Lr* genes confer adult plant resistance (APR). From this group, 7 genes (*Lr12*, *Lr13*, *Lr22a*/*b*, *Lr35*, *Lr37*, *Lr48* and *Lr49*) are race-specific and 8 (*Lr34*, *Lr46*, *Lr67*, *Lr68*, *Lr74*, *Lr75*, *Lr77* and *Lr78*) are race-non-specific, providing multipathogen, broad range resistance. *Lr12*, *Lr13* and *Lr22b* are associated with hypersensitive cell death. Race-non-specific APR against rusts attracts attention because it provides prolonged durability against the evolving pathogen population. The APR is expressed only in adult plants while the same genotype at the seedling stage is susceptible. Compared with the susceptible check, the adult plants with APR type of resistance show a longer latency period, lower score of infection type and smaller size of the uredinia [23].

Out of the 6 WAK genes (*TaWAK1*, *TaWAKL2*, *TaWAKL3*, *TaWAK4*, *TaWAKL1* and *TaWAKL2*) isolated from wheat, 2 genes (*TaWAK4* and *TaWAKL2*) encoded truncated proteins. The remaining genes encoded a set of domains typical for wall-associated kinases. Four genes (*TaWAK1*, *TaWAK2*, *TaWAK3* and *TaWAKL1*) were present as multiple copies in the wheat genome [24]. The *TaWAK6* gene was originally identified in our group in the wheat suppression subtractive hybridization (SSH) library as the short 291bp clone JG968933. The transcript level of the clone, in plants inoculated with *Puccinia triticina* spores, corresponded to the resistance of these plants. It was strongly induced in highly resistant lines (Tc*Lr9* and Tc*Lr26*), moderately in lines with intermediate resistance (Tc*Lr24* and Tc*Lr25*) and remained on very low level in the susceptible cultivar Thatcher (results not published). This study presents detailed characterization of the *TaWAK6*. It is a novel WAK-encoding gene in wheat with a role in quantitative, adult plant type of resistance against rust.

## Materials and methods

### Plant and pathogen material

The wheat cultivar Kontesa, cultivar Thatcher susceptible to leaf rust, lines Tc*Lr9*, Tc*Lr12*, Tc*Lr22*, Tc*Lr34* carrying *Lr* resistance genes and isogenic to susceptible cultivar Thatcher were studied. Wheat plants were grown in a growth chamber at 22°C, with a 16-h photoperiod and an illumination intensity of 60 μE m^-2^ s^-1^. A single spore isolate of *Puccinia triticina* (*Pt*) with an established avirulence/virulence formula [25] was used for plant inoculations. The primary seedling leaves and 3 leaves of adult plants, i.e. the flag leaf and 2 leaves below, were inoculated with urediniospores suspended (1 mg/ml) in water with Tween 20 using spray bottles (Roth). Inoculated plants were incubated for 24 hours in the dark at 18°C and 100% humidity and further cultivated in the same conditions as before inoculation [25].

### Identification and cloning of full cDNA of KR815340

The full cDNA sequence of the transcript originally identified as EST JG968933 [26] was obtained as a result of 5′3′-RACE extension with the 5′/3′ RACE Kit (Roche) according to the manual protocol using RNA from the Tc*Lr9* line isolated from leaves 4 days post inoculation with spores of *P*. *triticina* isolate.

### Identification of *TaWAK6* homeologs and phylogenic analysis

Genomic sequences of *T*. *aestivum* (IWGSC, INSDC Assembly, July 2018), *T*. *urartu* (INSDC Assembly GCA_000347455.1, Apr 2013) and the corresponding annotations (JSON format) were retrieved from the Ensembl Plants database [27]. Homeolog, ortholog and paralog sequences of *TaWAK6* (*TraesCS5B02G063600*; KR815340) were obtained from Ensembl Compara [28], a phylogeny-based orthology and paralogy prediction pipeline, which is integrated in the Ensembl Plants database. The inferred evolutionary relationships were also confirmed by graph-based orthology and prediction methods such as Reciprocal Best BLAST Hit (RBH) and Reciprocal Smallest Distance (RSD) [29]. TBLASTN was used (e-value < 10^−5^) to identify missing gene models in the annotations of genomic sequences of *Triticum* and *Aegilops* species. The candidate genome region determined by TBLASTN was extracted and used as the input in gene prediction program FGENESH [30]. Multiple protein sequence alignments of *TaWAK6* homeologs and orthologs were performed using MAFFT [31]. Maximum likelihood trees were built using MEGA7 [32] with the JTT model and 1000 bootstrap replicates. Tree topology was also supported by the neighbor-joining (NJ) method performed on alignments of full-length protein sequences as well as fragments corresponding to the kinase domain (InterPro Accession: IPR000719).

### *TaWAK6* overexpression vector construction and generation of transgenic wheat plants

cDNA of *TaWAK6* amplified with primers KR5_Gf and KR_kl2Re (S1 Table) was cloned in the *Sma*I site of pBRACT211 (John Innes Center, Norwich) between the maize *Ubi1* promoter and *nos* terminator. The *Asc*I-*Sph*I fragment (4695 bp) containing Ubi1:TaWAK6 cDNA and nos terminator was cloned into *Hind*II-*Sph*I sites of pGEM-3Z (Promega). The resultant pGEM3Z-*Ubi1*:*TaWAK6* was supplemented by ligating in the *Sma*I site the *Eco*RI-*Sca*I fragment from pCAMBIA3201 (CSIRO, Australia), which contained the 35S:*bar* gene for phosphinothricin selection. The final pGEM3Z-*Ubi*:*TaWAK6*-*35S*:*bar* vector (S1 Fig) was linearized with *Aat*II prior to gold particle loading and biolistic transformation. Biolistic transformation of the wheat cultivar Kontesa was done as described by Harwood, Ross [33] and Zalewski, Orczyk [34] and the plant regeneration was performed using a modified procedure as described by Binka, Orczyk [35]. Briefly, isolated immature wheat embryos pre-cultured for 1 day on MSB3 medium (Dicamba 3 mg/ml) were transferred to MSB3 with mannitol (72.9 g/l) and 4 hours later were subjected to transformation using Biolistic PDS-1000/He (Bio-Rad) with 1100 psi rupture discs. Gold microcarriers (1 μm diameter) were coated with DNA (5 μg/6 mg of microcarriers) of pGEM3Z-*Ubi*:*TaWAK6*-*35S*:*bar* plasmid linearized with *Aat*II in the presence of spermidine and CaCl_2_. The embryos were transferred to selection-free MSB6 medium (2,4-D 1 mg/ml, Picloram 2 mg/ml) 16 hours after biolistic transformation for 2 weeks in a dark room. Regenerating calli were transferred to MSB6 with phosphinothricin (2 ml/l) and cultured for 2 weeks under illumination. Regenerated plantlets, transferred to MIB medium (MSB6 without casein hydrolysate, ½ of Fe/EDTA, BAP 1 mg/ml, IAA 0.2 mg/ml) with phosphinothricin (2.5 ml/l) were cultured for a further 2 weeks. Rooted plants were cultured on ½MS with phosphinothricin (2.5 ml/l) and the bigger plants were transferred to pots filled with peat substrate. The presence of T-DNA was verified by PCR using gDNA as a template and bar 7/bar 8 primers (S1 Table).

### Nucleic acid isolation, reverse transcription and transcript quantification

Genomic DNA was isolated using a modified CTAB procedure. Total RNA was isolated from leaf samples ground in liquid N_2_ using TRI Reagent Solution (Invitrogen) according to the manufacturer’s instructions. RNA was treated with DNase (DNase I recombinant RNase-free; Roche) and Protector RNase inhibitor (Roche). RNA concentration and A260/A280 ratio were measured using a NanoDrop spectrophotometer (NanoDrop Technologies). First strand cDNA was synthesized with a random hexamer primer according to the manual protocol (RevertAid First Strand cDNA Synthesis Kit, Thermo Scientific). The *TaWAK6* transcript was quantified in 10 μl of PCR reaction mixture containing 1×Hot FIREPol EvaGreen qPCR Mix (Solis BioDyne), 0.4 μM of each primer: R_I_77ant, KinR_5nR [26] (S1 Table), and 1 μl of the template cDNA. Real-time PCR reactions were run in the Rotor-Gene Q cycler (Qiagen). Relative expression of *TaWAK6* was calculated by the two standard curves method with 18S rRNA [36] as a reference gene. Transcript quantitation was done in three biological replicates and each reaction was run in three technical repetitions.

### Histopathological analysis of wheat leaves inoculated with *Puccinia triticina* spores

Wheat leaves were inoculated using a sprayer with *Puccinia triticina* spores at a density of 1 mg/ml suspended in water with Tween 20. Infection types were evaluated on seedlings’ leaves 10 days post-inoculation (dpi) and on adult plants the first (flag), the second and the third leaves 16 dpi using the 0–4 scale by Roelfs and Martens [37]. Infection types were evaluated on the first, the second and the third leaves of nine plants from each tested line and Kontesa WT.

The latent period was established for the leaf fragment 4 cm long. Briefly, uredinia were counted each day starting from the first day after inoculation until the maximal number of uredinia was reached. The latent period value was calculated for each inoculated leaf (Eq 1) [38].

$$Latent\;period=t_{1}+(\frac{F}{2}-{nt}_{1})(t_{2}-t_{1})({nt}_{2}-{nt}_{1})$$ (1)

where F = final number of uredinia, t_1_ = day prior to 50% uredinia, t_2_ = day after 50% uredinia observed, nt_1_ = number of uredinia observed at t_1_, nt_2_ = number of uredinia observed at t_2_.

The length and the width of at least 20 uredinia per leaf were measured on the day after the latent period. The uredinium size (mm^2^) was calculated (Eq 2) [38].

$$Uredinium\;size=length\times width\times \frac{\pi }{4}$$ (2)

Inoculated leaves collected one day after the latent period were stained with calcofluor white to examine plant-pathogen interaction [25]. Briefly, leaf samples, cleared and fixed with ethanol/dichloromethane (3:1 v/v) with 0.15% trichloroacetic acid for 24 h, washed twice with 50% ethanol, twice with 0.05M sodium hydroxide, 3x in water and once with 0.1 M Tris–HCl, pH 8.5, were stained in calcofluor white (3.5 mg ml^−1^ in 0.1 M Tris–HCl, pH 9) and washed in water. The stained samples were examined under a fluorescence microscope (Nikon Diaphot, epifluorescence optics with excitation 340–380 nm, barrier filter 420 nm and dichroic mirror 400 nm) [25].

The number of all infection sites and the number of uredinia were quantified and the percentage of infection sites with uredinia was calculated (Eq 3) [25]

$$Percentage\;of\;infection\;sites\;with\;uredinia=\frac{number\;of\;infection\;sites\;with\;uredinia\times 100\%}{number\;of\;all\;infection\;sites}$$ (3)

The percentage of infection sites with uredinia, the average uredinium size and the average latent period were used to calculate the coefficient of resistance (Eq 4).

$$Coefficient\;of\;resistance=\frac{latent\;priod\;}{percentage\;of\;infection\;sites\;with\;uredinia\times uredinium\;size}$$ (4)

The latent period, the uredinium size and the percentage of infection sites with uredinia were evaluated on the first (flag) leaf using the three plants from each tested line and Kontesa WT.

### Statistical analysis

For statistical comparison of the relative expression of *TaWAK6*, the profiles of host-pathogen interaction, uredinia size and latent period, ANOVA followed by the least significant difference (LSD) post-hoc test was used (STATISTICA 10, StatSoft). For infection type analysis, the median, lower (25%) and upper (75%) quartile, minimum and maximum values were used, because variables are on the ordinal scale (qualitative scale) and the Kruskal-Wallis analysis of ranks was used (STATISTICA 10, StatSoft). Spearman correlation coefficients were calculated for the relative expression of *TaWAK6*, the scores of infection types on the first (flag), the second and the third leaves, the profiles of plant-pathogen interactions, the uredinium size and the latent period of the nontransgenic Kontesa and 7 transgenic lines.

## Results

### Characterization of *TaWAK6*

**Fig 1:**
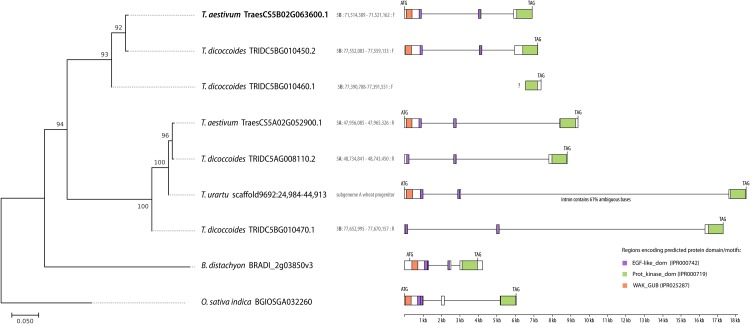
Phylogenic relationships among 8 putative *TaWAK6* (*TraesCS5B02G063600*; KR815340) homologs. *WAK6* is present uniquely in commelinids, with the exception of *Aegilops tauschii* and *Hordeum vulgare* (based on currently available genomic, transcriptomic and protein sequences). The phylogeny was reconstructed based on the amino acid sequence of kinase domains using the maximum likelihood method. The percentages of replicate trees in which the associated taxa clustered together in the bootstrap test (1000 replicates) are shown next to the branches. The tree was rooted with the sequences of two outgroup species: *Oryza sativa* and *Brachypodium distachyon*.

In the previous study we obtained the complete transcript sequence of KR815340 (*TraesCS5B02Go63600*.*1*) by RACE using cDNA of JG968933 and 5 other overlapping clones [26]. KR815340 (*TraesCS5B02Go63600*.*1*) encodes a 690-residue receptor-like protein with domain characteristics of the wall-associated kinase (WAK) family, including a cytoplasmic serine/threonine kinase domain, a calcium-binding epidermal growth factor (EGF_CA) domain and an extracellular galacturonan-binding (GUB) domain (Fig 1).

The protein sequence encoded by KR815340 corresponds (one substitution difference; S2 Fig) to the protein sequences of *TraesCS5B02G063600* transcript (S3 Fig) annotated in the recent genome assembly of *T*. *aestivum* (IWGSC, INSDC Assembly, Nov 2018). KR815340/*TraesCS5B02G063600* (henceforth *TraesCS5B02G063600*) is located, along with two homeo-paralogs (*TraesCS5B02G063500*, *TraesCS5B02G063700*), on the short arm of chromosome 5 of subgenome B. Both homeo-paralogs gene models are annotated by IWGSC with high confidence as nontranslating CDS (S2 Table). *TraesCS5B02G063600* is orthologous to the *TRIDC5BG010450* gene located on chromosome 5 of *T*. *dicoccoides*, the tetraploid emmer wheat (AABB), which, as reported, hybridized with *Aegilops tauschii* (the D-genome donor) to produce modern bread wheat [39] (Fig 1). In subgenome A, the gene corresponding to *TraesCS5B02G063600* is located on the short arm of chromosome 5A (*TraesCS5A02G052900*). This homeologous relationship is supported by the presence of the *TRIDC5AG008110* ortholog in subgenome A of *T*. *dicoccoides* as well as the *TraesCS5B02G063600* ortholog in *T*. *urartu* (Fig 1), the species that is reported to be the progenitor of wheat subgenome A [39]. We did not find homeologs of *TraesCS5B02G063600* in subgenome D of wheat, nor did we identify orthologs in *T*. *tauschii* (*Aegilops tauschii*), the species which, as reported by Pont and Salse [40] is the progenitor of subgenome D of wheat. Interestingly, the most similar protein (with a BLAST e-value of 2e-170) that could be found in currently available sequences for *Aegilops speltoides* is Snn1 (SnTox1), which was reported to be involved in biotrophic pathogen resistance [22].

The *TraesCS5B02G063600* gene and the closely related wheat gene *TraesCS5A02G052900* as well as orthologous genes in *T*. *dicoccoides*, *T*. *urartu*, *B*. *distachyon* (*BdWAK2*) and *O*. *sativa* reveal common gene structure (3 exons) as well as protein domain content and arrangement specific to WAK kinases (with the exception of *TRIDC5BG010460* and *TRIDC5AG008110*, whose truncated exon lacks the WAK-GUB domain) (Fig 1). On the basis of the presence or absence of arginine (R) adjacent to the catalytic aspartate (D) in the kinase domain, WAKs are classified into RD (arginine-aspartate) and non-RD (non-arginine-aspartate) kinases. Unlike *Arabidopsis* WAK proteins (encoding RD kinases), the *TraesCS5B02G063600* and its homologs encode the non-RD kinase (S4 Fig). Most of the pattern-recognition receptors (PRRs) that recognize conserved microbial or pathogen-associated molecular patterns are non-RD kinases [41]. Given that *TraesCS5B02G063600* represents all the key features of a WAK-encoding gene and chronologically it is the 6th wheat WAK-encoding gene, we assigned it as *TaWAK6*.

### Overexpression of *TaWAK6* elevates resistance against leaf rust in adult plants but not in seedlings

The 2896 immature embryos were transformed with the *TaWAK6* overexpression vector using the biolistic method. Out of 9 T0 plants selected on phosphinothricin-containing medium 7 were confirmed to have the *bar* gene. The plants from the T1-T2 generations with confirmed phosphinothricin resistance and the presence of the bar gene were self-pollinated and the seeds were used for the subsequent plant analyses.

#### Expression of *TaWAK6* in transgenic plants

**Fig 2:**
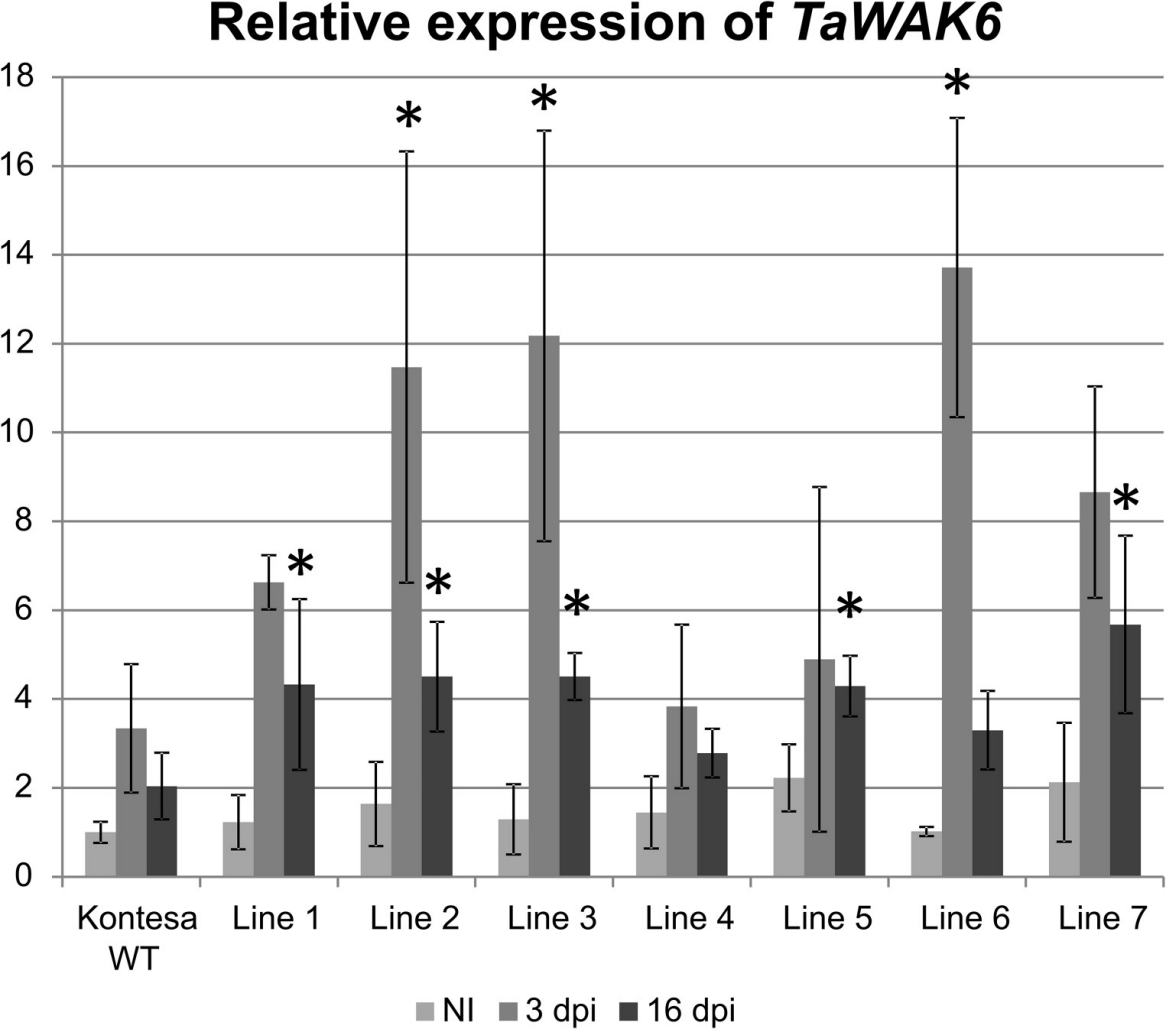
Relative expression of *TaWAK6* in flag leaves of non-transgenic wheat cultivar Kontesa WT and 7 transgenic lines overexpressing *TaWAK6* gene. NI—non-inoculated, 3 dpi, 16 dpi– 3, 16 days post-inoculation. The data represent mean values with standard deviation, asterisks indicate p < 0.05 using the ANOVA test followed by the LSD post-hoc test (STATISTICA 10, StatSoft).

Expression of *TaWAK6* was quantified in flag leaves of adult non-inoculated plants and in plants inoculated with a single spore isolate of *P*. *triticina* 3 and 16 days post-inoculation (dpi). Relative expression of *TaWAK6* in non-inoculated, non-transgenic Kontesa WT, assumed as 1.00, was used as a reference for the remaining relative expression values. The expression in WT Kontesa at 3 dpi and 16 dpi was 3.3 and 2.0 respectively. The pattern with a peak of expression at 3 dpi was a common feature of all lines (Fig 2). The values of relative expression varied in tested lines. In non-inoculated transgenic lines the values varied from 1.2 in line 1 to 2.2 in line 5. After inoculation the expression was induced and reached a peak at 3 dpi. The values were 6.6, 11.5, 12.2, 3.8, 4.9, 13.7 and 8.7 in lines 1, 2, 3, 4, 5, 6 and 7 respectively. Expression values at 16 dpi remained elevated but were lower than at 3 dpi (Fig 2).

#### Symptoms of infection on seedling and flag leaves

**Fig 3:**
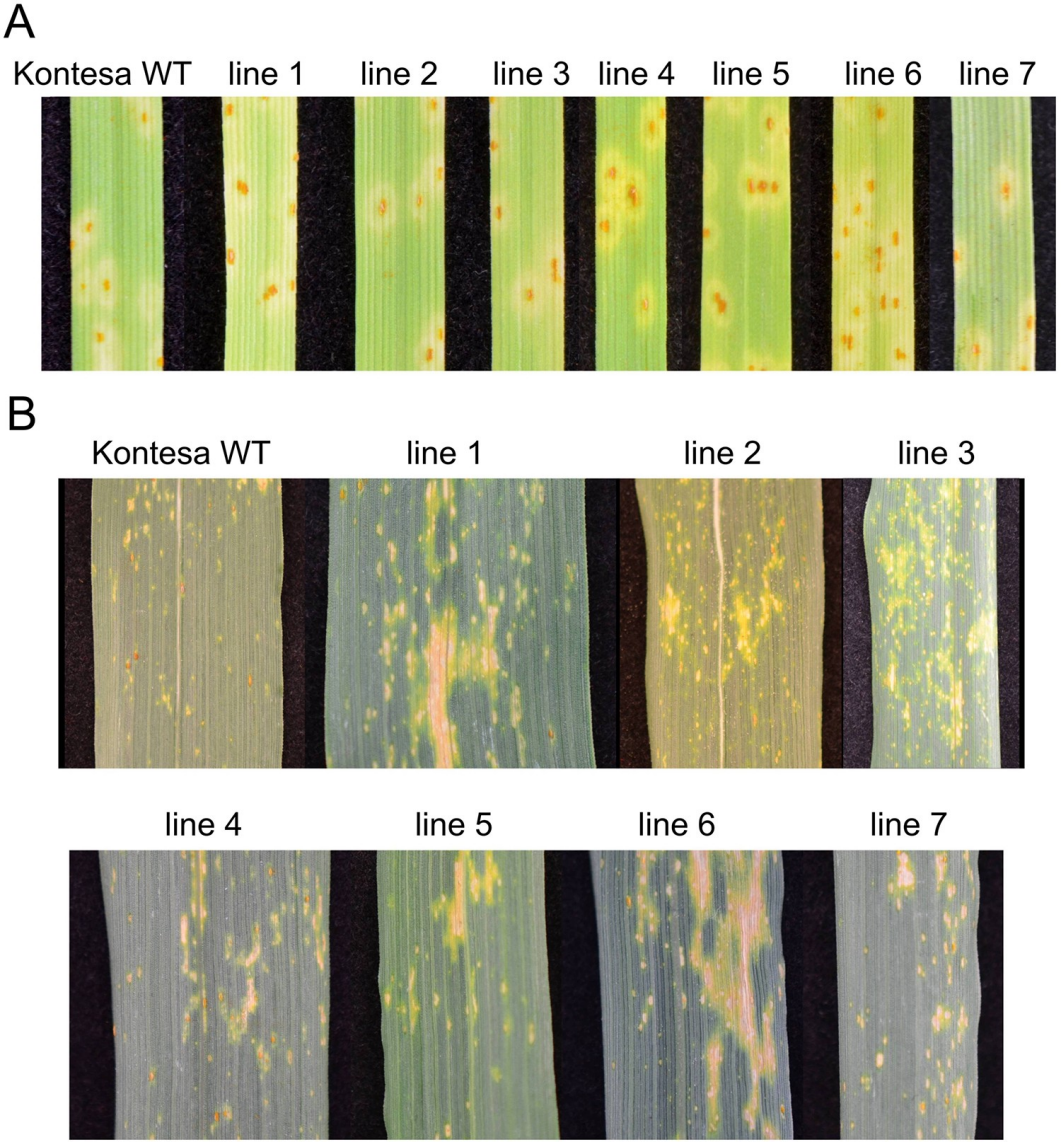
Infection symptoms on leaves of non-transgenic Kontesa and 7 transgenic lines overexpressing *TaWAK6* inoculated with single spore isolate of *P*. *triticina*. A—Seedling leaves 10 day post-inoculation (dpi). B—Adult plant, flag leaves, 16 dpi.

Seedlings of the cultivar Kontesa are susceptible to the used isolate of *P*. *triticina*. Seedlings of all transgenic lines overexpressing *TaWAK6* showed fully developed uredinia 10 days post-inoculation. The symptoms corresponded to infection type 3 according to the scale by Roelfs and Martens [37] was similar to nontransgenic cultivar Kontesa (Fig 3A).

Symptoms of infection on flag leaves of nontransgenic Kontesa showed no uredinia and small to medium sized uredinia surrounded by necrosis and/or chlorosis. The symptoms corresponded to a score of 0–3 according to the Roelfs and Martens [37] scale. Infection on flag leaves of transgenic lines with overexpression of *TaWAK6* was characterized by more micronecrotic reactions and fewer uredinia compared to nontransgenic Kontesa (Fig 3B). Both, the symptoms and the scores of infection types on flag leaves of transgenic plants indicated elevated resistance to leaf rust, which led to a more detailed analysis of plant-pathogen interactions in control plants and transgenic lines.

**Fig 4:**
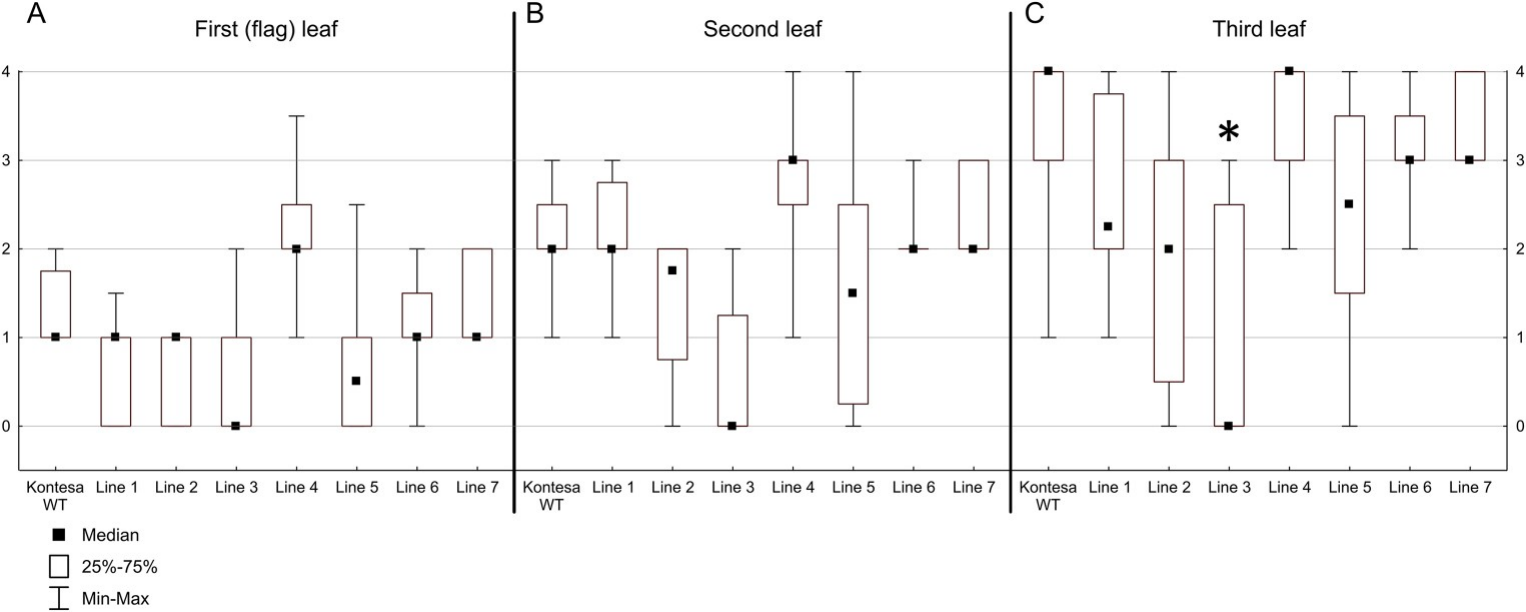
Infection types scored on the first (flag) leaf A, the second leaf B and on the third leaf C of nontransgenic Kontesa and 7 transgenic lines overexpressing *TaWAK6* gene inoculated with *P*. *triticina* single spore isolate.

Infection types were scored on the first (flag), the second and the third leaves. Since the results were qualitative they were presented as median, lower (25%) and upper (75%) quartiles and minimal and maximal scores (Fig 4). On the flag leaves for the non-transgenic Kontesa the median, the lower quartile and the minimal score were 1.0, the upper quartile was 1.75 and the maximal score was 2.0. For transgenic lines 1 and 2 the median was 1, the lower quartile was 0, and the upper quartile was 1. For transgenic line 3 the median, the lower quartile and the minimal score were 0, the upper quartile was 1 and the maximal score was 2.0. Transgenic line 4 was more susceptible than Kontesa; the median and the lower quartile were 2.0, the upper quartile was 2.5 and the maximal score was 3.5. For transgenic line 5 the median was 0.5, the lower quartile and the minimal score were 0, the upper quartile was 1 and the maximal score was 2.5. Lines 6 and 7 had the median, the lower and the upper quartile, and the maximal score very similar to non-transgenic Kontesa. The infection types in lines 1, 2, 3, and 5 showed a shift towards stronger quantitative resistance and in these lines there were leaves without uredinia (Fig 4A). On the second leaves for non-transgenic Kontesa, the median and the lower quartile were 2 and the upper quartile was 2.5. For transgenic lines 1, 6 and 7 the median was the same as Kontesa. For transgenic lines 2, 3 and 5 the median was lower than Kontesa; it was 1.75, 0 and 1.5 respectively. For transgenic line 4 the median was 3 (Fig 4B). On the third leaves for non-transgenic Kontesa and for transgenic line 4 the median was 4. For the other lines the median was lower than for Kontesa; it was 2.25, 2, 0, 2.5, 3 and 3 (for lines 1, 2, 3, 5, 6 and 7 respectively) (Fig 4C).

#### Percentage of infection sites with uredinia

**Fig 5:**
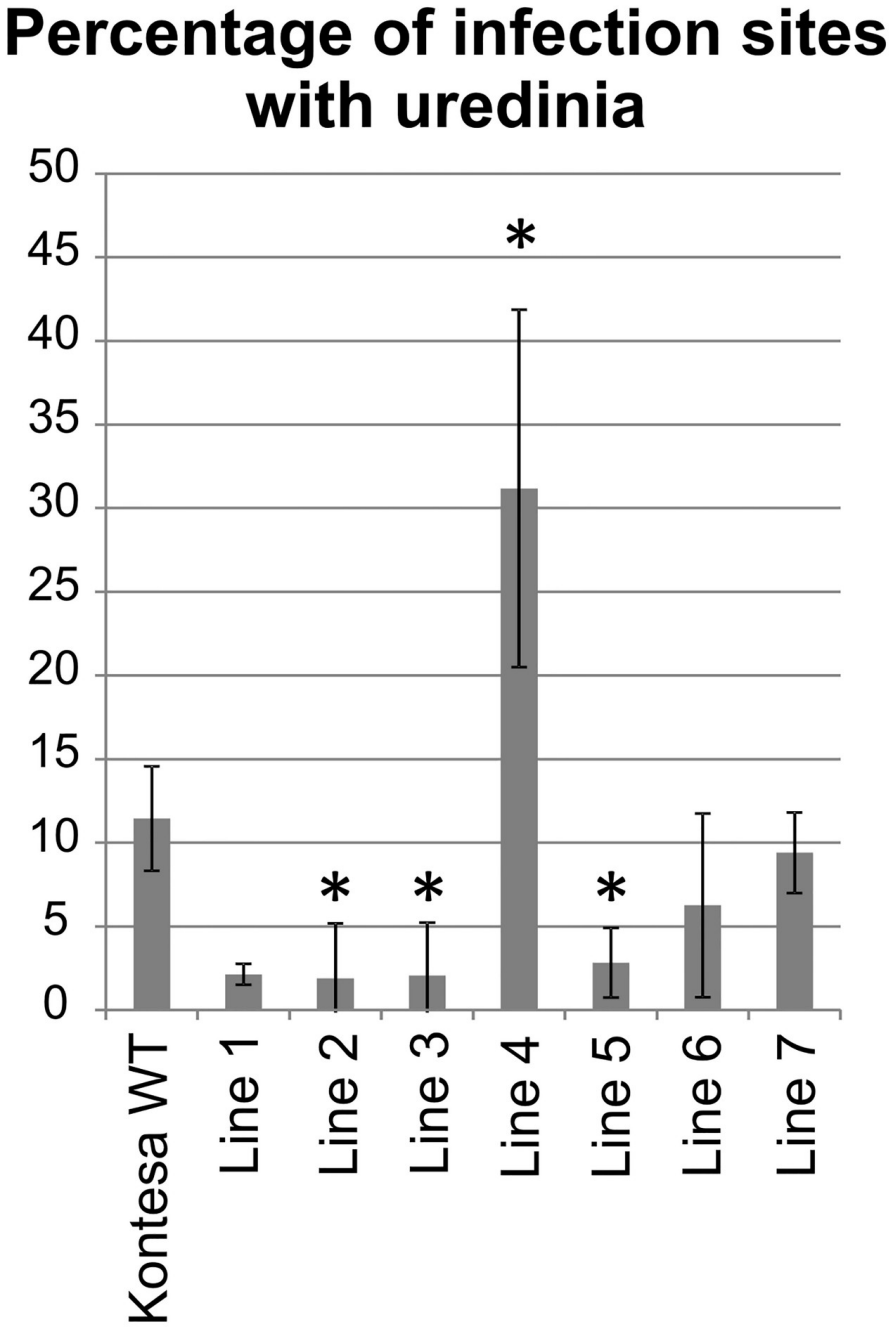
Percentage of infection sites with uredinia scored on flag leaves of non-transgenic Kontesa and 7 transgenic lines overexpressing *TaWAK6* gene 16 days post inoculation with *P*. *triticina*. Data represent mean values with standard errors of the mean; asterisk indicates p < 0.05 using the ANOVA test followed by LSD post-hoc test (STATISTICA 10, StatSoft).

The percentage of infection sites with uredinia was calculated on flag leaves. In non-transgenic Kontesa it was 11.4%. In lines 2, 3, 4, 5 and 6 the percentage (2.1%, 1.9%, 2.1%, 2.8% and 6.3% respectively) was lower compared to Kontesa. In line 7 the value 9.4% was in the range found in Kontesa. The value in line 4 (31.2%) was much higher than in WT Kontesa (Fig 5).

#### Uredinia size and latent period

**Fig 6:**
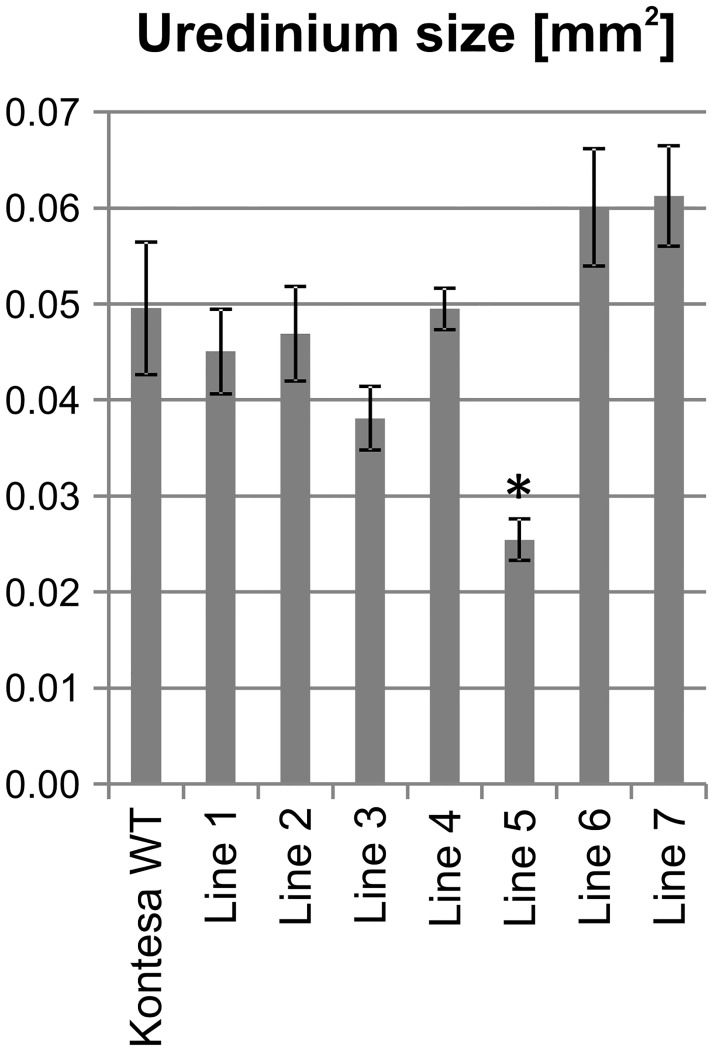
The average uredinia size developed on flag leaves of non-transgenic Kontesa and seven transgenic lines overexpressing *TaWAK6* gene 16 days post inoculation with *P*. *triticina*. Data represent mean values with standard errors of the mean; asterisk indicates p < 0.05 using the ANOVA test followed by LSD post-hoc test (STATISTICA 10, StatSoft).

The average uredinia size developed on flag leaves of non-transgenic Kontesa was 0.05 mm^2^. Uredinia developed on flag leaves of transgenic lines 1, 2, 3 and 4 were smaller, the average size was 0.045, 0.047, 0.038 and 0.025 mm^2^ respectively. The uredinia size on line 4 was in the range found on non-transgenic Kontesa. The uredinia developed on lines 6 and 7 were bigger than in non-transgenic Kontesa with the average size 0.06 and 0.061 mm^2^ (Fig 6).

**Fig 7:**
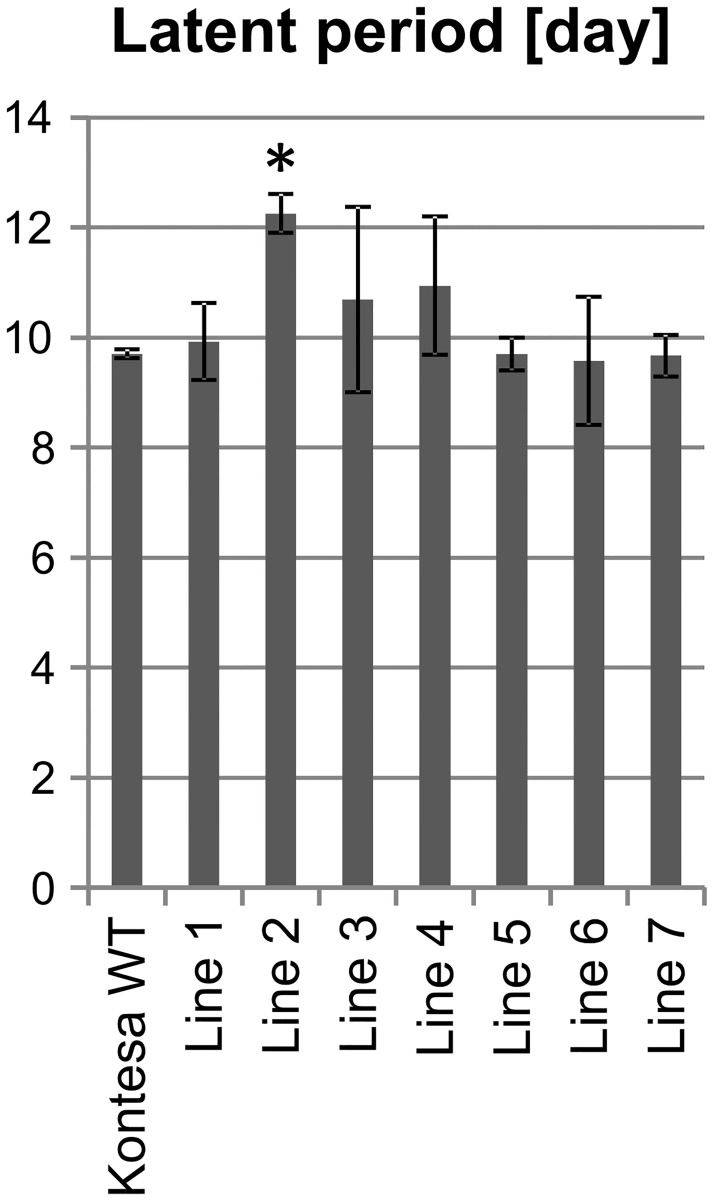
Latent period of *P*. *triticina* development on flag leaves of non-transgenic wheat cultivar Kontesa and 6 transgenic lines overexpressing *TaWAK6* gene 16 days post inoculation with *P*. *triticina*. The data represent mean values with standard deviations; the asterisk indicates p < 0.05 using the ANOVA test followed by LSD post-hoc test (STATISTICA 10, StatSoft).

The latent period, defined as time needed to develop half of the final number of uredinia, was analyzed. It is considered that a longer latent period and associated with this slower pathogen development indicate stronger quantitative resistance. The latent period observed on flag leaves of the non-transgenic Kontesa was 9.7 days. The latent periods on transgenic lines 1, 2, 3 and 4 were 9.92, 12.25, 10.69 and 10.94 days respectively. The value observed for lines 5, 6 and 7 was similar to WT Kontesa (Fig 7).

#### Coefficient of resistance

**Fig 8:**
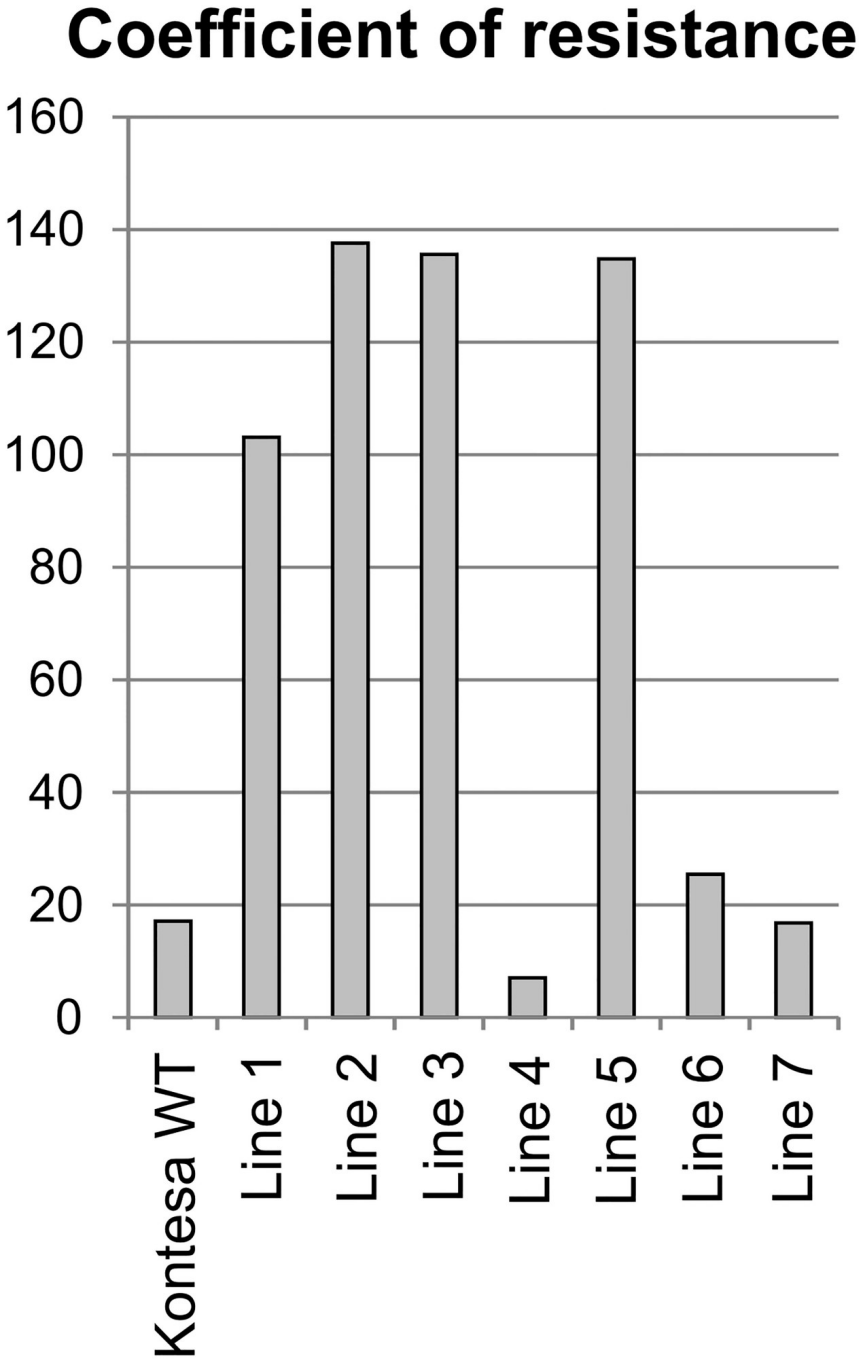
The coefficient of resistance in transgenic *TaWAK6* lines and non-transgenic Kontesa. The values were calculated using Eq 4 and the results of the latent period, the percentage of infection sites with uredinia and the uredinium size.

The coefficient of resistance was calculated using the latent period, the percentage of infection sites with uredinia and the uredinia size. The coefficient takes into account the values of the three parameters important in quantitative APR and it allows for a complex comparison of the transgenic lines with the non-transgenic Kontesa. The coefficient of resistance calculated for WT Kontesa was 17.1. The values of the coefficient were very high in transgenic lines 1, 2, 3 and 5 and they equaled 103.1, 137.6, 135.6 and 134.8 respectively. In line 6 it was 25.4 while in line 7 it was 16.8 similar as in WT Kontesa. The coefficient in line 4 was 7.1, much lower than in WT Kontesa (Fig 8).

#### Correlation coefficients

Spearman correlation coefficients (*r*_*S*_) were calculated for the relative expression of *TaWAK6*, 3 and 16 dpi, the scores of infection types on the first (flag), the second and the third leaves, the percentage of infection sites with uredinia, the uredinium size and the latent period of the non-transgenic Kontesa and 7 transgenic lines (Table 1). The relative expression 3 and 16 dpi of *TaWAK6* showed medium negative correlations with the infection types scored on the first (flag), the second and the third leaf. The *r*_*S*_ values were −0.69 (p≤0.005) and −0.70 (p≤0.0005) for the flag leaf, -0.59 (p≤0.005) and -0.62 (p≤0.001) for the second leaf and -0.64 (p≤0.005) for the third leaf. Relative expression 16 dpi of *TaWAK6* showed medium negative correlations *r*_*S*_ = −0.57 (p≤0.005) with the percentage of infection sites with uredinia. The infection type scored on the first (flag) leaf showed a strong positive correlation with the infection type on the second leaf, *r*_*S*_ = 0.75 (p≤0.001), and a medium correlation, *r*_*S*_ = 0.58 (p≤0.001), with the third leaf. The infection type scored on the flag leaf was strongly correlated with the percentage of infection sites with uredinia *r*_*S*_ = 0.85 (p≤0.001), with uredinium size, *r*_*S*_ = 0.72 (p≤0.001) and the latent period, *r*_*S*_ = 0.68 (p≤0.005).

**Table 1:** Spearman correlation coefficients (*r*_*s*_) were calculated for the relative expression of *TaWAK6*, the scores of infection types, the percentage of infection sites with uredinia, the uredinium size and the latent period on flag leaves of nontransgenic Kontesa and 7 transgenic lines. The correlation coefficient (STATISTICA 10, StatSoft) was significant at p<0.05. *r*_*s*_ values between 0 and |0.29| were defined as a weak correlation, values between |0.3| and |0.69| were defined as medium, and values between |0.7| and |1| were defined as a strong correlation.

			Relative expression of *TaWAK6*		Infection type			Percentage of infection sites with uredinia	Uredinium size	Latent period
			3 dpi	16 dpi	1st (flag) leaf	2nd leaf	3rd leaf			
Relative expression of *TaWAK6*	3 dpi	r_s_	1.00	1.00	**-0.69**	**-0.59**	-0.30		-0.50	
		p			0.003	0.004	0.177		0.667	
	16 dpi	r_s_	1.00	1.00	**-0.70**	**-0.62**	**-0.64**	**-0.57**	-0.30	-0.13
		p			0.000	0.001	0.001	0.004	0.140	0.581
Infection type	1st (flag) leaf	r_s_	**-0.69**	**-0.70**	1.00	**0.75**	**0.58**	**0.85**	**0.72**	**0.68**
		p	0.003	0.000		0.000	0.000	0.000	0.000	0.003
	2nd leaf	r_s_	**-0.59**	**-0.62**	**0.75**	1.00	**0.69**	**0.85**	**0.68**	**0.54**
		p	0.004	0.001	0.000		0.000	0.000	0.000	0.024
	3rd leaf	r_s_	-0.30	**-0.64**	**0.58**	**0.69**	1.00	**0.76**	**0.57**	**0.61**
		p	0.177	0.001	0.000	0.000		0.000	0.007	0.013
Percentage of infection sites with uredinia		r_s_		**-0.57**	**0.85**	**0.85**	**0.76**	1.00	**0.65**	**0.62**
		p		0.004	0.000	0.000	0.000		0.002	0.011
Uredinium size		r_s_	-0.50	-0.30	**0.72**	**0.68**	**0.57**	**0.65**	1.00	0.29
		p	0.667	0.140	0.000	0.000	0.007	0.002		0.209
Latent period		r_s_		-0.13	**0.68**	**0.54**	**0.61**	**0.62**	0.29	1.00
		p		0.581	0.003	0.024	0.013	0.011	0.209	

The percentage of infection sites with uredinia showed a medium correlation with the uredinium size *r*_*S*_ = 0.65 (p≤0.005) and the latent period *r*_*S*_ = 0.62 (p≤0.05) indicating that inoculation of transgenic *TaWAK6* plants led to fewer uredinia, which were smaller and with longer development. The average uredinium size on the flag leaf was strongly correlated with the scores of infection type, *r*_*S*_ = 0.72 (p≤0.0001), indicating that bigger uredinia correlated with higher Roelfs and Martens [37] scores. Both parameters were typical for weaker plant resistance. Compared with the flag leaf, the values of this correlation for the second (*r*_*S*_ = 0.68, p≤0.0001) and the third leaf (*r*_*S*_ = 0.57, p≤0.01) were lower. This indicated that the flag leaf showed the strongest resistance, which reflected the key feature of the adult plant resistance.

## Discussion

In this study we characterized a WAK-encoding gene with a function in wheat resistance against *P*. *triticina*. WAKs are the only class of receptor protein kinases which provide a molecular link between the extracellular space and the cytoplasm. Their molecular model established in *Arabidopsis* shows that their recognition of pectin or pectin degradation products (the former released upon pathogen infection) trigger developmental or defense signaling pathways respectively [4, 42]. Consistently with the model, WAKs were reported to function in plant resistance against pathogens [8, 9, 11, 19, 22, 43], developmental processes [44] and responses to environmental factors [45]. T*aWAK6* (TraesCS5B01G063600; KR815340) is the 6th member of WAK-encoding genes identified in wheat (S2 Table). Originally, it was cloned as a strongly expressed EST in the Tc*Lr9* isogenic line upon inoculation with *P*. *triticina* spores [26]. *TaWAK6*-encoded protein contains all typical WAK domains: galacturonan-binding GUB, epidermal growth factor-like EGF, EGF-calcium binding EGF-CA, transmembrane region TR and Ser/Thr kinase. The number and the pattern of these domains correspond very well to the protein structure encoded by previously reported *TaWAK* genes (S5 Fig). This group consists of 4 *WAK* and 2 *WAKL* genes identified by Liu, Liu [24], the *WAK5* gene reported by Yang, Qi [21], the WAK-encoding *Snn1* gene cloned and characterized by Shi, Zhang [22], 3 genes–*Stb6* (*WAKL4*), *WAKL2* and *WAKL2* –reported by Saintenac, Lee (19) and W5G2U8_WHEAT, a potential WAK receptor protein [20] (S2 Table). *TaWAK6* shares common gene structure of 3 exons with all wheat homeologs.

Localization of *TaWAK6* paralogs in *T*. *urartu* (AA) and *T*. *dicoccoides* (AABB) genomes is consistent with the evolution of bread wheat. *T*. *urartu* is reported to provide genome A to the allotetraploid (AABB) wild emmer *T*. *dicoccoides*, which in turn is the progenitor of the A and B subgenomes of allohexaploid (AABBDD) bread wheat [39]. The closest homology to *TaWAK6* homeo-ortholog TraesCS5A02G052900 (subgenome A of wheat) showed the scaffold 9692:24,984–44,913 in *T*. *urartu* (AA) and TRIDC5AG008110 localized on chromosome 5 in subgenome B of *T*. *dicoccoides* (AABB). Homology of *TaWAK6* (chromosome 5B of wheat) with two paralogs on chromosome 5B of *T*. *dicoccoides* further confirms the consistency of *TaWAK6* paralogs’ localization in both subgenomes of wheat and its wild emmer progenitor. The lack of *TaWAK6* paralogs in *Aegilops tauschii* (DD), the progenitor of wheat subgenome D, provides a compatible phylogenic interpretation of the lack of *TaWAK6* homeo-orthologs in the D subgenome of wheat. *TaWAK6*, its homeologs in wheat and orthologs in *T*. *dicoccoides*, *T*. *urartu*, *B*. *distachyon* (*BdWAK2*) and *O*. *sativa* showed common structure of the genes (3 exons) and protein domain content and arrangement specific to WAK kinases.

Integration of the *TaWAK6* transgene under the *Ubi1* promoter in the wheat genome led to cumulative expression of the *TaWAK6* native gene and the *Ubi1*::*TaWAK6* construct (S1 Fig). The cumulative expression of *TaWAK6* did not affect seed germination, plant growth or development of transgenic wheat. The expression peak of *TaWAK6* in flag leaves of cv. Kontesa non-transgenic control and transgenic lines was observed 3 dpi (Fig 2). A similar expression pattern was found in *P*. *triticina* inoculated seedlings carrying strong leaf rust resistance genes *Lr9* and *Lr26* [25, 26]. Although the levels of *TaWAK6* cumulative expression varied among the individual transgenic lines, the pattern of temporal regulation was similar. Relative expression in lines 2, 3 and 6 was strongly up-regulated, expression in lines 1, 5 and 7 was moderately elevated, and expression in line 4 was similar to the non-transgenic control. Such differences of transgene expression are common in independently regenerated transgenic plants and could be the result of the diverse structure of integrated DNA molecules and different integration loci. In this particular case the diversity of expression provided another variable which was included in the statistical analyses.

Strong up-regulation of the transgene upon pathogen infection, observed in 6 transgenic lines, indicated that the *Ubi1* promoter, commonly used as a strong constitutive promoter, showed a strong pathogen-dependent activity. The result, although unexpected, was compatible with other reports showing that *Ubi1*-driven transgenes were induced by the environmental stresses heat, freezing and drought [46–48] and biotic factors–*Rhizoctonia cerealis* and *Bipolaris sorokiniana* infections [47, 48]. Our results further confirm that *Ubi1* characteristics do not fully comply with the constitutive promoter definition.

Seedlings of cv. Kontesa were susceptible to the *P*. *triticina* isolate used in the study and the cumulative *TaWAK6* expression in the transgenic seedlings did not affect this. However, it did affect quantitative resistance of adult plants against the same pathogen. Moreover, infection scored on the flag (the 1st) leaf of lines 1, 2, 3 and 5 indicated higher resistance compared to the non-transgenic Kontesa. Similar results were observed on the 2nd and 3rd leaves (Fig 4). Infection scores observed on the 1st, the 2nd and the 3rd leaves showed a strong to medium correlation with the cumulative *TaWAK6* expression in these leaves (Table 1).

The quantitative nature of adult plants partial resistance (PR) was further characterized by the percentage of infection sites with uredinia, the uredinium size and the latent period. These three components of APR were evaluated in transgenic lines and they also showed a medium to high correlation with *TaWAK6* expression. The percentage of infection sites with uredinia was much lower in line 1, 2, 3 and 5 than in WT Kontesa. Uredinia that developed on lines 1, 2, 3 and 5 were smaller compared to WT Kontesa, and the latent period in lines 1, 2, 3, and 4 was longer than in WT Kontesa (Figs 5, 6 and 7 respectively). Similar characteristics were observed in lines with known APR genes: *Lr12*, *Lr22* and *Lr34* [49–51]. In the Tc*Lr12* line (isogenic to susceptible cv. Thatcher) the percentage of infection sites with uredinia was lower than in cv. Thatcher. Compared with Thatcher, the latent period of this line was significantly longer and uredinia were much smaller. In Tc*Lr22*, the quantitative resistance relied on significantly smaller uredinia. The APR of the Tc*Lr34* line was associated with a lower percentage of infection sites with uredinia. Uredinia were significantly smaller, with a significantly longer latent period (S6–S8 Figs). Despite different characteristics of PR components, the observed lower percentage of infection sites with uredinia, smaller uredinia and longer latent periods in isogenic lines compared to Thatcher were similar and consistent for all of these lines. According to Herrera-Foessel, Singh [52], all of them were important because their cumulative effect reduced the epidemic rate in the field even though the individual plants supported full life cycle of the pathogen.

Characteristics of the three transgenic lines 1, 2 and 3 with significantly higher *TaWAK6* expression were, in this respect, similar to isogenic lines with the APR type of resistance. The conclusion is further strengthened by the observed medium to strong negative correlation between the *TaWAK6* cumulative expression and the scores of infection type in transgenic lines (Table 1). It is also worth adding that the correlation of expression and infection scores was strong in the flag leaf (*r*_*S*_ = -0.70; p≤0.0001), while similar correlations in 2nd and 3rd leaves had medium values (*r*_*S*_ = -0.62; p≤0.0005). Similar observations of smaller uredinia and a longer latent period in APR were reported by Hoogkamp, Chen [53], Kloppers and Pretorius [54] and Soleiman, Solis [55]. Singh and Huerta-Espino [51] reported that differences in latent periods between the isolines increased from the 4-leaf stage onwards and stabilized by the 5-leaf stage, which was in agreement with our findings showing that the older plant (i.e. flag leaf) showed stronger resistance compared to 2nd and 3rd leaves. According to Kloppers and Pretorius [54], the longer latent period and smaller uredinia in lines with APR *Lr13*, *Lr34* and *L37* genes indicated improved field resistance against wheat rust. Measurement of the latent period of the pathogen allowed for the accurate estimation of pre-haustorial barley resistance against *Puccinia hordei* [53]. The coefficient of resistance introduced in our research took into account the three important parameters of PR and allowed discrimination of the quantitative differences between the individual lines (Fig 8).

## Supporting information

S1 Fig The final pGEM3Z-*Ubi*:*TaWAK6-35S*:*bar* vector. (TIF)

S2 Fig Protein alignment between KR815340 and TraesCS5B02G063600. (PDF)

S3 Fig cDNA sequence alignment between KR815340 and TraesCS5B02G063600. (PDF)

S4 Fig Clustal Omega alignment of the closest WAK proteins. The kinase active site is highlighted in red. The blue hash sign indicates the arginine and non-arginine residues adjacent to the active site aspartic acid, RD and non RD respectively.
(PDF)

S5 Fig Protein motifs and domains of TaWAK proteins. Protein motifs and domains of TaWAK proteins identified using the Simple Modular Architecture Research Tool (SMART) database (http://smart.embl-heidelberg.de/).
(TIF)

S6 Fig Percentage of infection sites with uredinia. Percentage of infection sites with uredinia scored on flag leaves of susceptible Thatcher and isogenic lines Tc*Lr12*, Tc*Lr22* and Tc*Lr34* 16 days post inoculation with single spore isolate of *P*. *triticina*. Genes *Lr12*, *Lr22* and *Lr34* confer adult plant resistance (APR) of wheat against leaf rust.
(TIF)

S7 Fig Latent period of leaf rust. Latent period of leaf rust scored on flag leaves of susceptible cultivar Thatcher and isogenic lines Tc*Lr12*, Tc*Lr22* and Tc*Lr34* inoculated with single spore isolate of *Puccinia triticina*. Genes *Lr12*, *Lr22* and *Lr34* confer adult plant resistance (APR) of wheat against leaf rust.
(TIF)

S8 Fig Uredinia size. Uredinia size scored on flag leaves of susceptible cultivar Thatcher and isogenic lines Tc*Lr12*, Tc*Lr22* and Tc*Lr34* 6 days post inoculation with *Puccinia triticina* single spores isolate. Genes *Lr12*, *Lr22* and *Lr34* confer adult plant resistance (APR) of wheat against leaf rust.
(TIF)

S1 Table Primers and reaction conditions for PCR. (PDF)

S2 Table S2 List of *TaWAK* and *TaWAK-like* genes. (PDF)
