# Supplementary figures and images for: TaWAK6 encoding wall-associated kinase is involved in wheat resistance to leaf rust similar to adult plant resistance
Source: PLoS One. 2020 Jan 13;15(1):e0227713. doi: 10.1371/journal.pone.0227713 (PMC6957155; doi:10.1371/journal.pone.0227713)

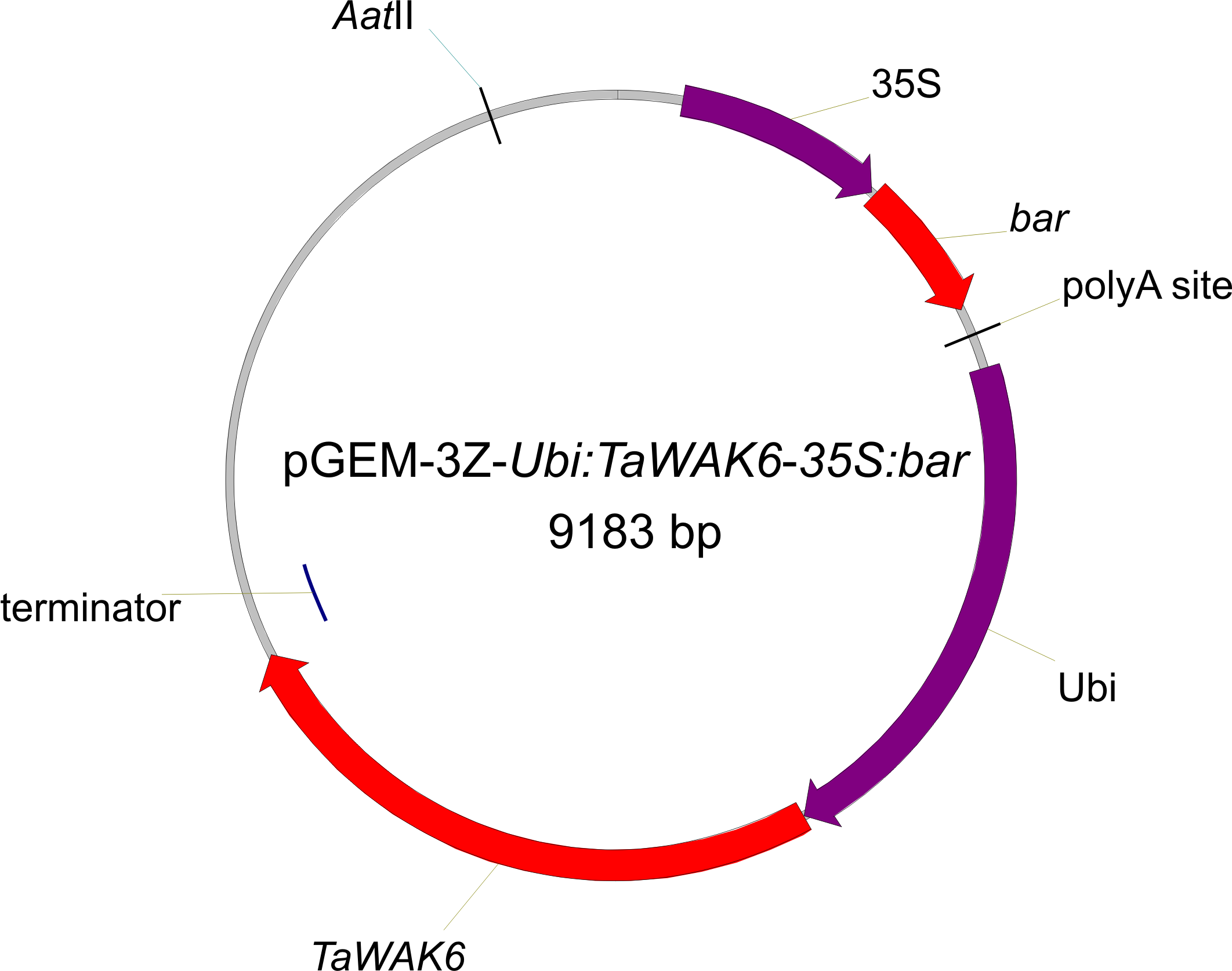

Supplement: S1 Fig — (TIF) [file pone.0227713.s001.tif]

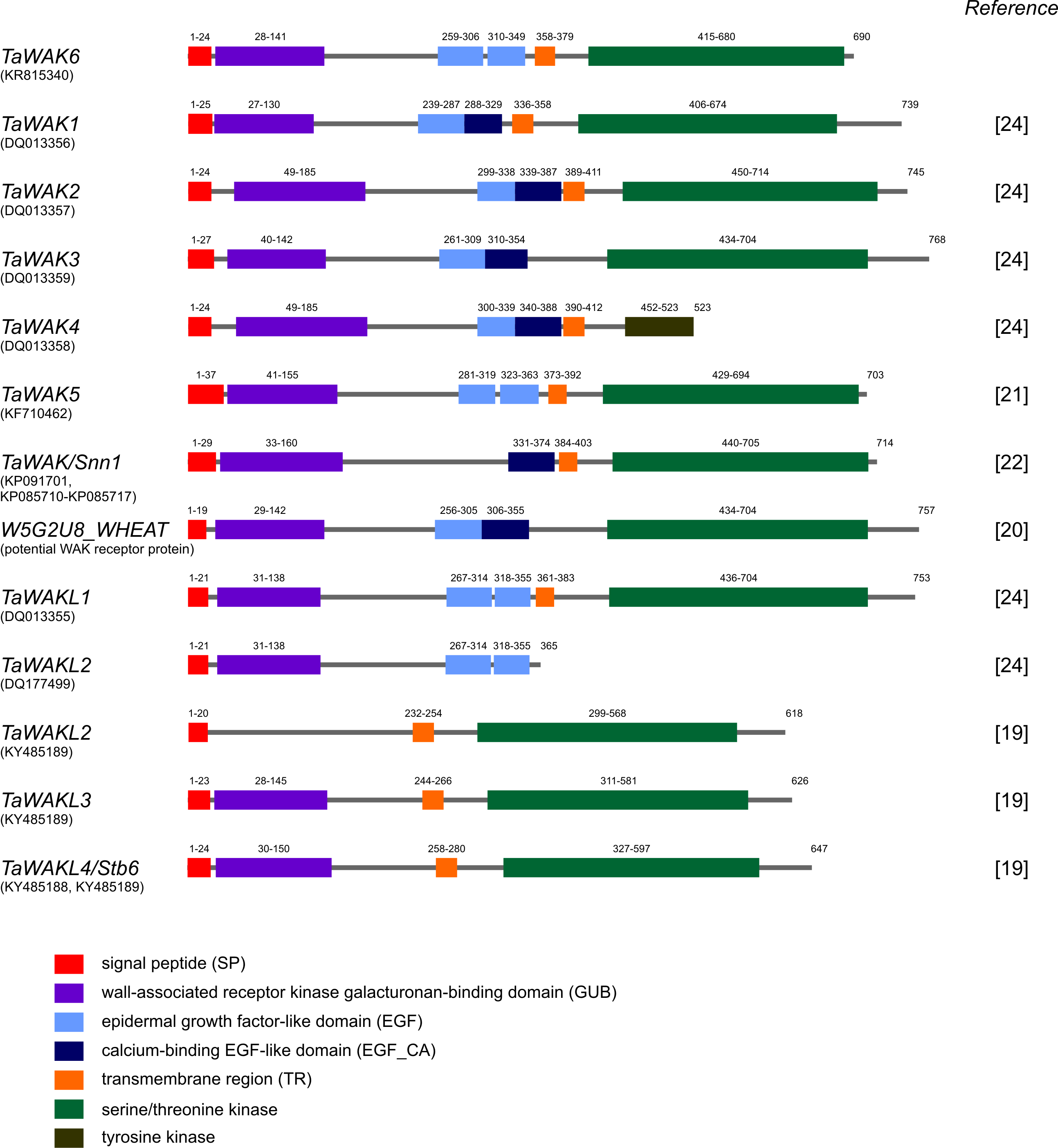

Supplement: S5 Fig — Protein motifs and domains of TaWAK proteins identified using the Simple Modular Architecture Research Tool (SMART) database (http://smart.embl-heidelberg.de/). (TIF) [file pone.0227713.s005.tif]

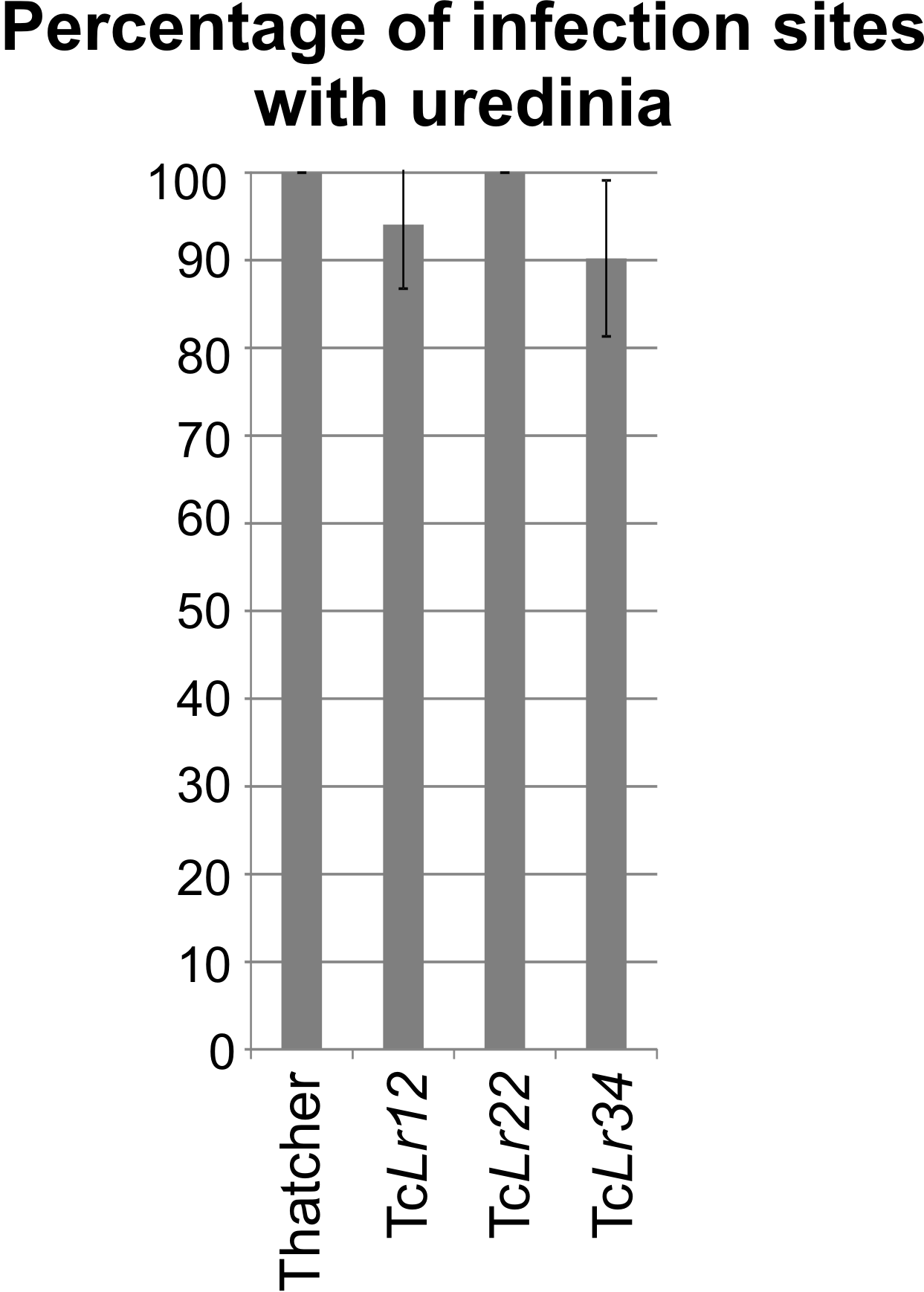

Supplement: S6 Fig — Percentage of infection sites with uredinia scored on flag leaves of susceptible Thatcher and isogenic lines TcLr12, TcLr22 and TcLr34 16 days post inoculation with single spore isolate of P. triticina. Genes Lr12, Lr22 and Lr34 confer adult plant resistance (APR) of wheat against leaf rust. (TIF) [file pone.0227713.s006.tif]

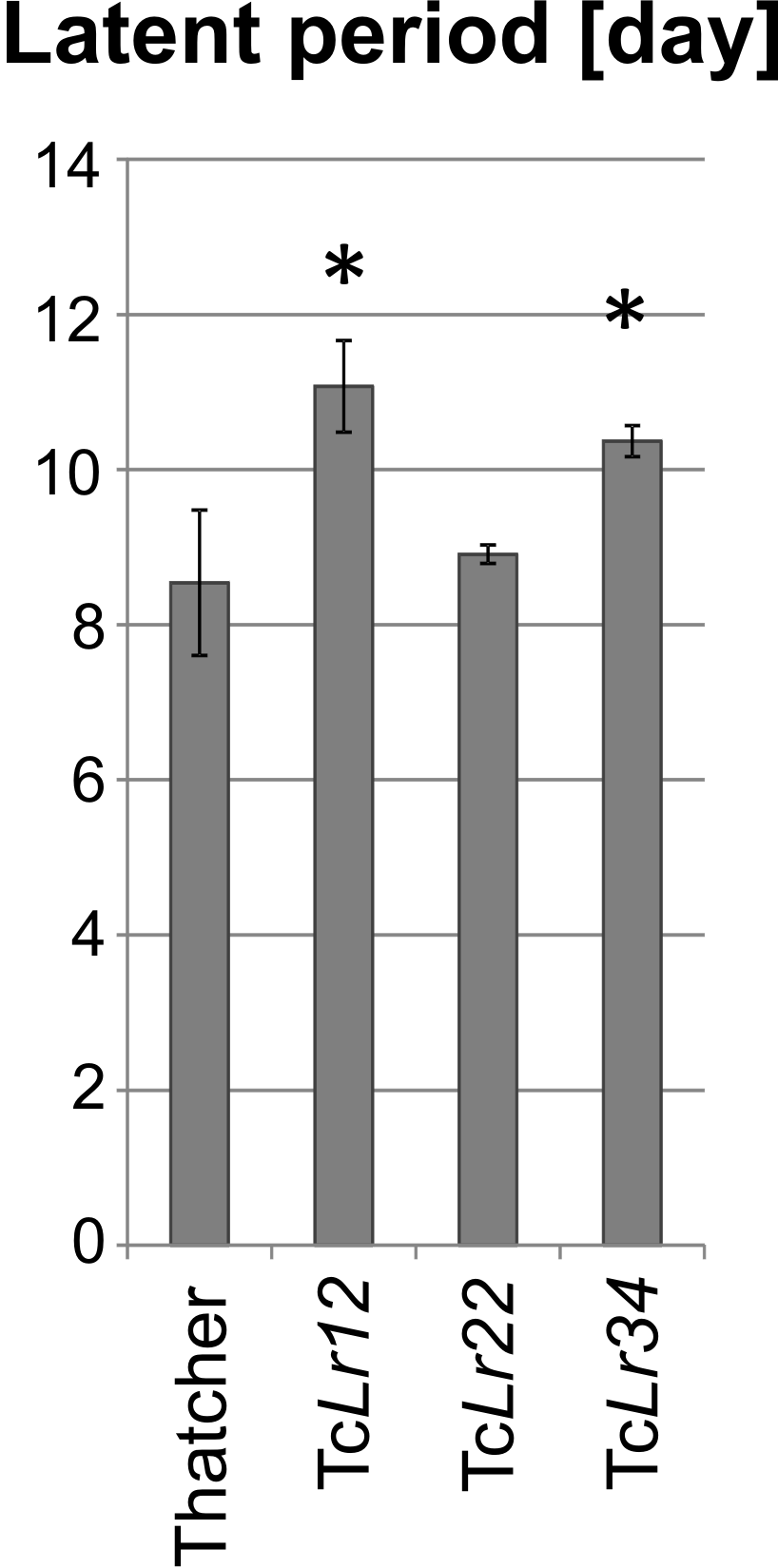

Supplement: S7 Fig — Latent period of leaf rust scored on flag leaves of susceptible cultivar Thatcher and isogenic lines TcLr12, TcLr22 and TcLr34 inoculated with single spore isolate of Puccinia triticina. Genes Lr12, Lr22 and Lr34 confer adult plant resistance (APR) of wheat against leaf rust. (TIF) [file pone.0227713.s007.tif]

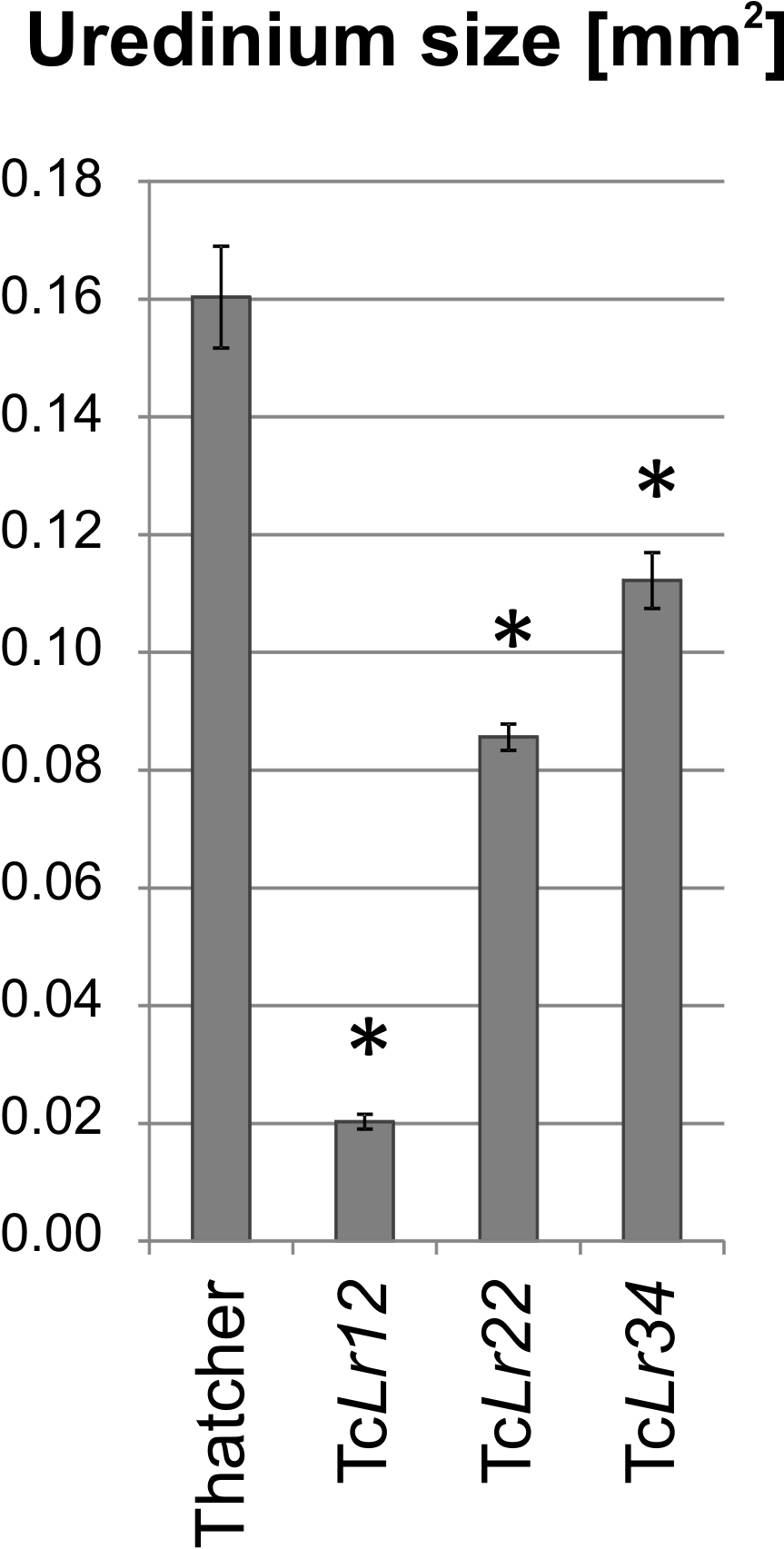

Supplement: S8 Fig — Uredinia size scored on flag leaves of susceptible cultivar Thatcher and isogenic lines TcLr12, TcLr22 and TcLr34 6 days post inoculation with Puccinia triticina single spores isolate. Genes Lr12, Lr22 and Lr34 confer adult plant resistance (APR) of wheat against leaf rust. (TIF) [file pone.0227713.s008.tif]
