# Supplementary material for: TaWAK6 encoding wall-associated kinase is involved in wheat resistance to leaf rust similar to adult plant resistance
Source: PLoS One. 2020 Jan 13;15(1):e0227713. doi: 10.1371/journal.pone.0227713 (PMC6957155; doi:10.1371/journal.pone.0227713)
Supplement: S2 Fig — (PDF) [file pone.0227713.s002.pdf]

```
########################################
# Program: needle
# Rundate: Tue 18 Dec 2018 18:55:42
# Commandline: needle
#    -auto
#    -stdout
#    -asequence emboss_needle-I20181218-185541-0426-29813692-p1m.asequence
#    -bsequence emboss_needle-I20181218-185541-0426-29813692-p1m.bsequence
#    -datafile EBLOSUM62
#    -gapopen 10.0
#    -gapextend 0.5
#    -endopen 10.0
#    -endextend 0.5
#    -aformat3 pair
#    -sprotein1
#    -sprotein2
# Align_format: pair
# Report_file: stdout
########################################

#=======================================
#
# Aligned_sequences: 2
# 1: KR815340
# 2: TraesCS5B02G063600.1
# Matrix: EBLOSUM62
# Gap_penalty: 10.0
# Extend_penalty: 0.5
#
# Length: 690
# Identity:     689/690 (99.9%)
# Similarity:   689/690 (99.9%)
# Gaps:           0/690 ( 0.0%)
# Score: 3693.0
#
#
#=======================================

KR815340           1 MSRTFQLLLVLALVGIVRVSGSRAHKNVTHGPHSCSGVDVPYPFGIVEDG     50
                     ||||||||||||||||||||||||||||||||||||||||||||||||||
TraesCS5B02G0      1 MSRTFQLLLVLALVGIVRVSGSRAHKNVTHGPHSCSGVDVPYPFGIVEDG     50

KR815340          51 GGGDYRAGFHVMCDAGEPVLHTTGGDGKPVKIGNFSIQAAEARVWLPVVW    100
                     ||||||||||||||||||||||||||||||||||||||||||||||||||
TraesCS5B02G0     51 GGGDYRAGFHVMCDAGEPVLHTTGGDGKPVKIGNFSIQAAEARVWLPVVW    100

KR815340         101 QCYDSSGKPSRSDYRNLEFNKGGVYRISNAKNNLFVLGCKTTGYLASQPD    150
                     ||||||||||||||||||||||||||||||||||||||||||||||||||
TraesCS5B02G0    101 QCYDSSGKPSRSDYRNLEFNKGGVYRISNAKNNLFVLGCKTTGYLASQPD    150

KR815340         151 QGSGESTSYAQFTGCLCYCNNSQSAVNGACSGVGCCHVDIPPDLTDNWVA    200
                     ||||||||||||||||||||||||||||||||||||||||||||||||||
TraesCS5B02G0    151 QGSGESTSYAQFTGCLCYCNNSQSAVNGACSGVGCCHVDIPPDLTDNWVA    200

KR815340         201 FMSYDHTDKVNFSPCDYAFVAEKKHYTFNTTDLKRALRQNTWGWEMPVVL    250
                     ||||||||||||||||||||||||||||||||||||||||||||||||||
TraesCS5B02G0    201 FMSYDHTDKVNFSPCDYAFVAEKKHYTFNTTDLKRALRQNTWGWEMPVVL    250

KR815340         251 DWAIRDSPTCKEARKKEGYACISSNSLCLNSTNGPGYICNCRRGYEGNLY    300
                     ||||||||||||||||||||||||||||||||||||||||||||||||||
TraesCS5B02G0    251 DWAIRDSPTCKEARKKEGYACISSNSLCLNSTNGPGYICNCRRGYEGNLY    300

KR815340         301 IVDGCTDINECEHLDHYSCKGVCTNRQGSYECTCPKHTHSADPYKEVCSP    350
                     ||||||||||||||||||||||||||||||||||||||||||||||||||
TraesCS5B02G0    301 IVDGCTDINECEHLDHYSCKGVCTNRQGSYECTCPKHTHSADPYKEVCSP    350

KR815340         351 NFPTNAKIIVGAIGGLLVMVIMVFFWLLIEEKRKMKEHFEKVGGPTLEKL    400
                     |||||||||||||||||||||||||||||||||||||||||.||||||||
TraesCS5B02G0    351 NFPTNAKIIVGAIGGLLVMVIMVFFWLLIEEKRKMKEHFEKNGGPTLEKL    400

KR815340         401 NNIKLFKKEDIRKIQKSSNIIGSGGFGKVYKGCIGDNNELVAVKEPINVN    450
                     ||||||||||||||||||||||||||||||||||||||||||||||||||
TraesCS5B02G0    401 NNIKLFKKEDIRKIQKSSNIIGSGGFGKVYKGCIGDNNELVAVKEPINVN    450

KR815340         451 SANKGQFANEIIIQSPVIHRNIVKLVGCCLQVEVPILVYEFVPNGSLHDI    500
                     ||||||||||||||||||||||||||||||||||||||||||||||||||
TraesCS5B02G0    451 SANKGQFANEIIIQSPVIHRNIVKLVGCCLQVEVPILVYEFVPNGSLHDI    500

KR815340         501 LHNGSRMHLDMCKRLKIAAESAEGLAYMHSKTTTTILHGDVKPANILLND    550
                     ||||||||||||||||||||||||||||||||||||||||||||||||||
TraesCS5B02G0    501 LHNGSRMHLDMCKRLKIAAESAEGLAYMHSKTTTTILHGDVKPANILLND    550

KR815340         551 EFTPKISDFGISRLIVTDMQHTGNVIGDMSYMDPVLLQTGLLTKKSDVYS    600
                     ||||||||||||||||||||||||||||||||||||||||||||||||||
TraesCS5B02G0    551 EFTPKISDFGISRLIVTDMQHTGNVIGDMSYMDPVLLQTGLLTKKSDVYS    600

KR815340         601 FGVVLLELITRKKASHSDKNSLLRNFLDAYTKDKSVIELVDKELAEVDRE    650
                     ||||||||||||||||||||||||||||||||||||||||||||||||||
TraesCS5B02G0    601 FGVVLLELITRKKASHSDKNSLLRNFLDAYTKDKSVIELVDKELAEVDRE    650

KR815340         651 ILDNLGEMIMQCLNLDVNQRPEMTDVAERLRDMVKRFNAQ    690
                     ||||||||||||||||||||||||||||||||||||||||
TraesCS5B02G0    651 ILDNLGEMIMQCLNLDVNQRPEMTDVAERLRDMVKRFNAQ    690


#---------------------------------------
#---------------------------------------
```

S2 Fig. Protein alignment between KR815340 and TraesCS5B02G063600.
