# Supplementary material for: TaWAK6 encoding wall-associated kinase is involved in wheat resistance to leaf rust similar to adult plant resistance
Source: PLoS One. 2020 Jan 13;15(1):e0227713. doi: 10.1371/journal.pone.0227713 (PMC6957155; doi:10.1371/journal.pone.0227713)
Supplement: S3 Fig — (PDF) [file pone.0227713.s003.pdf]

```
########################################
# Program: needle
# Rundate: Tue 18 Dec 2018 19:01:06
# Commandline: needle
#    -auto
#    -stdout
#    -asequence emboss_needle-I20181218-190104-0278-83315858-p1m.asequence
#    -bsequence emboss_needle-I20181218-190104-0278-83315858-p1m.bsequence
#    -datafile EDNAFULL
#    -gapopen 10.0
#    -gapextend 0.5
#    -endopen 10.0
#    -endextend 0.5
#    -aformat3 pair
#    -snucleotide1
#    -snucleotide2
# Align_format: pair
# Report_file: stdout
########################################

#=======================================
#
# Aligned_sequences: 2
# 1: KR815340.1
# 2: TraesCS5B02G063600.1
# Matrix: EDNAFULL
# Gap_penalty: 10.0
# Extend_penalty: 0.5
#
# Length: 2473
# Identity:    2071/2473 (83.7%)
# Similarity:  2071/2473 (83.7%)
# Gaps:         400/2473 (16.2%)
# Score: 10347.0
#
#
#=======================================

KR815340.1         1 ACACAACACGTTGATGAGAATGCATGTTCAAGCCCAACGCCTTAGCTATT     50
                                                                       
TraesCS5B02G0      1 --------------------------------------------------      0

KR815340.1        51 TAAACACTTGAAGGGTCGTTGTGAGATCACTGCATATCGAAAAGTGAGAG    100
                                                                       
TraesCS5B02G0      1 --------------------------------------------------      0

KR815340.1       101 CGCCACGATCGACCGGGAGTAGCGATGTCGAGGACATTTCAGTTGCTGCT    150
                                             ||||||||||||||||||||||||||
TraesCS5B02G0      1 ------------------------ATGTCGAGGACATTTCAGTTGCTGCT     26

KR815340.1       151 AGTTCTTGCACTAGTAGGAATAGTTAGAGTGAGTGGTTCCCGCGCTCATA    200
                     ||||||||||||||||||||||||||||||||||||||||||||||||||
TraesCS5B02G0     27 AGTTCTTGCACTAGTAGGAATAGTTAGAGTGAGTGGTTCCCGCGCTCATA     76

KR815340.1       201 AGAACGTCACCCACGGCCCGCACAGCTGCAGCGGCGTGGATGTCCCCTAC    250
                     ||||||||||||||||||||||||||||||||||||||||||||||||||
TraesCS5B02G0     77 AGAACGTCACCCACGGCCCGCACAGCTGCAGCGGCGTGGATGTCCCCTAC    126

KR815340.1       251 CCGTTCGGCATCGTGGAGGATGGCGGCGGTGGCGACTACCGCGCTGGGTT    300
                     ||||||||||||||||||||||||||||||||||||||||||||||||||
TraesCS5B02G0    127 CCGTTCGGCATCGTGGAGGATGGCGGCGGTGGCGACTACCGCGCTGGGTT    176

KR815340.1       301 CCATGTCATGTGTGACGCCGGTGAGCCGGTACTGCACACCACCGGCGGCG    350
                     ||||||||||||||||||||||||||||||||||||||||||||||||||
TraesCS5B02G0    177 CCATGTCATGTGTGACGCCGGTGAGCCGGTACTGCACACCACCGGCGGCG    226

KR815340.1       351 ACGGAAAGCCCGTTAAGATCGGCAACTTCTCCATCCAGGCGGCTGAGGCC    400
                     ||||||||||||||||||||||||||||||||||||||||||||||||||
TraesCS5B02G0    227 ACGGAAAGCCCGTTAAGATCGGCAACTTCTCCATCCAGGCGGCTGAGGCC    276

KR815340.1       401 CGCGTGTGGCTGCCTGTAGTGTGGCAGTGCTACGACTCCTCCGGCAAGCC    450
                     ||||||||||||||||||||||||||||||||||||||||||||||||||
TraesCS5B02G0    277 CGCGTGTGGCTGCCTGTAGTGTGGCAGTGCTACGACTCCTCCGGCAAGCC    326

KR815340.1       451 GAGTAGGTCAGACTACAGGAACCTAGAGTTCAACAAAGGGGGCGTGTACC    500
                     ||||||||||||||||||||||||||||||||||||||||||||||||||
TraesCS5B02G0    327 GAGTAGGTCAGACTACAGGAACCTAGAGTTCAACAAAGGGGGCGTGTACC    376

KR815340.1       501 GCATCTCCAACGCCAAGAACAACCTCTTTGTCCTGGGCTGCAAAACCACG    550
                     ||||||||||||||||||||||||||||||||||||||||||||||||||
TraesCS5B02G0    377 GCATCTCCAACGCCAAGAACAACCTCTTTGTCCTGGGCTGCAAAACCACG    426

KR815340.1       551 GGCTACCTCGCGAGCCAGCCGGACCAGGGCTCCGGCGAAAGCACGTCTTA    600
                     ||||||||||||||||||||||||||||||||||||||||||||||||||
TraesCS5B02G0    427 GGCTACCTCGCGAGCCAGCCGGACCAGGGCTCCGGCGAAAGCACGTCTTA    476

KR815340.1       601 CGCCCAGTTCACCGGCTGCCTCTGCTACTGCAACAACTCCCAGAGCGCGG    650
                     ||||||||||||||||||||||||||||||||||||||||||||||||||
TraesCS5B02G0    477 CGCCCAGTTCACCGGCTGCCTCTGCTACTGCAACAACTCCCAGAGCGCGG    526

KR815340.1       651 TGAACGGTGCCTGCTCCGGGGTAGGCTGCTGCCACGTAGACATTCCGCCG    700
                     ||||||||||||||||||||||||||||||||||||||||||||||||||
TraesCS5B02G0    527 TGAACGGTGCCTGCTCCGGGGTAGGCTGCTGCCACGTAGACATTCCGCCG    576

KR815340.1       701 GACCTCACCGACAACTGGGTGGCCTTCATGAGCTACGACCACACCGACAA    750
                     ||||||||||||||||||||||||||||||||||||||||||||||||||
TraesCS5B02G0    577 GACCTCACCGACAACTGGGTGGCCTTCATGAGCTACGACCACACCGACAA    626

KR815340.1       751 GGTCAACTTCAGCCCCTGCGATTATGCGTTTGTGGCGGAGAAAAAACATT    800
                     ||||||||||||||||||||||||||||||||||||||||||||||||||
TraesCS5B02G0    627 GGTCAACTTCAGCCCCTGCGATTATGCGTTTGTGGCGGAGAAAAAACATT    676

KR815340.1       801 ACACCTTCAACACCACCGACCTCAAGAGGGCACTGCGCCAGAACACCTGG    850
                     ||||||||||||||||||||||||||||||||||||||||||||||||||
```

```
TraesCS5B02G0     677 ACACCTTCAACACCACCGACCTCAAGAGGGCACTGCGCCAGAACACCTGG    726

KR815340.1        851 GGCTGGGAGATGCCGGTGGTCCTCGACTGGGCCATCCGCGACAGCCCCAC    900
                      ||||||||||||||||||||||||||||||||||||||||||||||||||
TraesCS5B02G0     727 GGCTGGGAGATGCCGGTGGTCCTCGACTGGGCCATCCGCGACAGCCCCAC    776

KR815340.1        901 CTGCAAGGAGGCAAGGAAGAAGGAGGGGTACGCCTGCATAAGCTCCAACA    950
                      ||||||||||||||||||||||||||||||||||||||||||||||||||
TraesCS5B02G0     777 CTGCAAGGAGGCAAGGAAGAAGGAGGGGTACGCCTGCATAAGCTCCAACA    826

KR815340.1        951 GTCTCTGCCTCAACTCTACCAATGGACCTGGATACATCTGCAACTGCCGT   1000
                      ||||||||||||||||||||||||||||||||||||||||||||||||||
TraesCS5B02G0     827 GTCTCTGCCTCAACTCTACCAATGGACCTGGATACATCTGCAACTGCCGT    876

KR815340.1       1001 CGAGGCTACGAGGGCAACCTCTATATTGTTGATGGTTGTACCGATATAAA   1050
                      ||||||||||||||||||||||||||||||||||||||||||||||||||
TraesCS5B02G0     877 CGAGGCTACGAGGGCAACCTCTATATTGTTGATGGTTGTACCGATATAAA    926

KR815340.1       1051 TGAGTGTGAGCATCTTGACCACTACTCTTGCAAGGGAGTGTGCACGAACA   1100
                      ||||||||||||||||||||||||||||||||||||||||||||||||||
TraesCS5B02G0     927 TGAGTGTGAGCATCTTGACCACTACTCTTGCAAGGGAGTGTGCACGAACA    976

KR815340.1       1101 GACAAGGTTCTTACGAATGTACATGCCCCAAACATACACACAGTGCTGAT   1150
                      ||||||||||||||||||||||||||||||||||||||||||||||||||
TraesCS5B02G0     977 GACAAGGTTCTTACGAATGTACATGCCCCAAACATACACACAGTGCTGAT   1026

KR815340.1       1151 CCGTACAAAGAAGTATGCAGCCCGAATTTTCCAACCAACGCAAAGATCAT   1200
                      ||||||||||||||||||||||||||||||||||||||||||||||||||
TraesCS5B02G0    1027 CCGTACAAAGAAGTATGCAGCCCGAATTTTCCAACCAACGCAAAGATCAT   1076

KR815340.1       1201 TGTAGGTGCAATAGGGGGCCTGTTAGTTATGGTAATTATGGTCTTCTTCT   1250
                      ||||||||||||||||||||||||||||||||||||||||||||||||||
TraesCS5B02G0    1077 TGTAGGTGCAATAGGGGGCCTGTTAGTTATGGTAATTATGGTCTTCTTCT   1126

KR815340.1       1251 GGCTTCTTATTGAAGAGAAAAGAAAGATGAAAGAACATTTTGAAAAGGTT   1300
                      |||||||||||||||||||||||||||||||||||||||||||||||..|
TraesCS5B02G0    1127 GGCTTCTTATTGAAGAGAAAAGAAAGATGAAAGAACATTTTGAAAAGAAT   1176

KR815340.1       1301 GGCGGACCTACATTGGAAAAGCTTAATAATATAAAGCTATTCAAAAAGGA   1350
                      ||||||||||||||||||||||||||||||||||||||||||||||||||
TraesCS5B02G0    1177 GGCGGACCTACATTGGAAAAGCTTAATAATATAAAGCTATTCAAAAAGGA   1226

KR815340.1       1351 AGATATCAGGAAAATTCAGAAAAGTAGCAACATTATTGGAAGTGGTGGTT   1400
                      ||||||||||||||||||||||||||||||||||||||||||||||||||
TraesCS5B02G0    1227 AGATATCAGGAAAATTCAGAAAAGTAGCAACATTATTGGAAGTGGTGGTT   1276

KR815340.1       1401 TTGGTAAGGTTTACAAGGGGTGCATTGGGGATAATAACGAGTTAGTTGCA   1450
                      ||||||||||||||||||||||||||||||||||||||||||||||||||
TraesCS5B02G0    1277 TTGGTAAGGTTTACAAGGGGTGCATTGGGGATAATAACGAGTTAGTTGCA   1326

KR815340.1       1451 GTGAAGGAGCCCATCAATGTTAACTCAGCAAACAAGGGTCAATTTGCAAA   1500
                      ||||||||||||||||||||||||||||||||||||||||||||||||||
TraesCS5B02G0    1327 GTGAAGGAGCCCATCAATGTTAACTCAGCAAACAAGGGTCAATTTGCAAA   1376

KR815340.1       1501 TGAGATCATCATTCAGTCTCCAGTCATTCACAGGAACATTGTGAAGCTCG   1550
                      ||||||||||||||||||||||||||||||||||||||||||||||||||
TraesCS5B02G0    1377 TGAGATCATCATTCAGTCTCCAGTCATTCACAGGAACATTGTGAAGCTCG   1426

KR815340.1       1551 TAGGCTGTTGCTTACAAGTTGAAGTCCCAATCTTGGTCTATGAGTTTGTA   1600
                      ||||||||||||||||||||||||||||||||||||||||||||||||||
TraesCS5B02G0    1427 TAGGCTGTTGCTTACAAGTTGAAGTCCCAATCTTGGTCTATGAGTTTGTA   1476

KR815340.1       1601 CCTAATGGTAGCCTTCATGACATTCTCCATAATGGTAGCAGGATGCATCT   1650
                      ||||||||||||||||||||||||||||||||||||||||||||||||||
TraesCS5B02G0    1477 CCTAATGGTAGCCTTCATGACATTCTCCATAATGGTAGCAGGATGCATCT   1526

KR815340.1       1651 CGATATGTGTAAACGTCTAAAAATTGCTGCTGAATCGGCGGAAGGTCTAG   1700
                      ||||||||||||||||||||||||||||||||||||||||||||||||||
TraesCS5B02G0    1527 CGATATGTGTAAACGTCTAAAAATTGCTGCTGAATCGGCGGAAGGTCTAG   1576

KR815340.1       1701 CTTATATGCATTCAAAAACCACTACCACAATCCTCCATGGTGATGTTAAA   1750
                      ||||||||||||||||||||||||||||||||||||||||||||||||||
TraesCS5B02G0    1577 CTTATATGCATTCAAAAACCACTACCACAATCCTCCATGGTGATGTTAAA   1626

KR815340.1       1751 CCAGCTAATATACTTCTGAATGACGAGTTCACACCAAAGATCTCAGACTT   1800
                      ||||||||||||||||||||||||||||||||||||||||||||||||||
TraesCS5B02G0    1627 CCAGCTAATATACTTCTGAATGACGAGTTCACACCAAAGATCTCAGACTT   1676

KR815340.1       1801 TGGTATATCAAGGTTAATTGTTACAGATATGCAACACACTGGAAATGTTA   1850
                      ||||||||||||||||||||||||||||||||||||||||||||||||||
TraesCS5B02G0    1677 TGGTATATCAAGGTTAATTGTTACAGATATGCAACACACTGGAAATGTTA   1726

KR815340.1       1851 TTGGTGACATGAGTTATATGGATCCAGTTCTTCTACAAACAGGATTACTA   1900
                      ||||||||||||||||||||||||||||||||||||||||||||||||||
TraesCS5B02G0    1727 TTGGTGACATGAGTTATATGGATCCAGTTCTTCTACAAACAGGATTACTA   1776

KR815340.1       1901 ACCAAGAAGAGTGATGTCTATAGTTTTGGAGTTGTGCTCTTGGAGCTCAT   1950
                      ||||||||||||||||||||||||||||||||||||||||||||||||||
TraesCS5B02G0    1777 ACCAAGAAGAGTGATGTCTATAGTTTTGGAGTTGTGCTCTTGGAGCTCAT   1826

KR815340.1       1951 TACAAGGAAGAAGGCATCACATTCTGACAAGAATAGCTTATTGAGGAACT   2000
                      ||||||||||||||||||||||||||||||||||||||||||||||||||
TraesCS5B02G0    1827 TACAAGGAAGAAGGCATCACATTCTGACAAGAATAGCTTATTGAGGAACT   1876

KR815340.1       2001 TTCTTGATGCTTACACGAAGGACAAGTCTGTGATTGAGCTCGTGGACAAG   2050
                      ||||||||||||||||||||||||||||||||||||||||||||||||||
TraesCS5B02G0    1877 TTCTTGATGCTTACACGAAGGACAAGTCTGTGATTGAGCTCGTGGACAAG   1926

KR815340.1       2051 GAACTTGCAGAGGTAGATCGAGAGATTCTTGATAATCTAGGAGAGATGAT   2100
                      ||||||||||||||||||||||||||||||||||||||||||||||||||
TraesCS5B02G0    1927 GAACTTGCAGAGGTAGATCGAGAGATTCTTGATAATCTAGGAGAGATGAT   1976

KR815340.1       2101 TATGCAATGTCTAAATCTTGATGTCAATCAGAGACCGGAGATGACAGATG   2150
                      ||||||||||||||||||||||||||||||||||||||||||||||||||
```

```
TraesCS5B02G0   1977 TATGCAATGTCTAAATCTTGATGTCAATCAGAGACCGGAGATGACAGATG   2026

KR815340.1      2151 TTGCAGAGCGTCTTCGTGACATGGTTAAGAGGTTTAATGCCCAATAGAAT   2200
                     |||||||||||||||||||||||||||||||||||||||||||||||
TraesCS5B02G0   2027 TTGCAGAGCGTCTTCGTGACATGGTTAAGAGGTTTAATGCCCAATAG---   2073

KR815340.1      2201 GATCAACTGGATGATCATGTGGTTTCGCAGTAAGTTGATGAATTTTACAG   2250

TraesCS5B02G0   2074 --------------------------------------------------   2073

KR815340.1      2251 AATGACGGAAGTATTATGCATTTATTTGCTTGTGCGCTTATTTTGCGTAG   2300

TraesCS5B02G0   2074 --------------------------------------------------   2073

KR815340.1      2301 ATAAAAATACTGTGAAAGAAGTATGGTACATATATATTTCCACACATATG   2350

TraesCS5B02G0   2074 --------------------------------------------------   2073

KR815340.1      2351 GTAGATTTCCTTAGAGATCATATATATTTCCACACATGGGACATGTGGAA   2400

TraesCS5B02G0   2074 --------------------------------------------------   2073

KR815340.1      2401 TTGTTTGCCCGAATAAATTGTTTTGTGTATTTTGTATGTTCTTCTTGTAA   2450

TraesCS5B02G0   2074 --------------------------------------------------   2073

KR815340.1      2451 TTTGTGGTTGTTTAACATGAATG   2473

TraesCS5B02G0   2074 -----------------------   2073


#--------------------------------------
#--------------------------------------
```

S3 Fig. cDNA sequence alignment between KR815340 and TraesCS5B02G063600 (KR815340).
