# Supplementary material for: TaWAK6 encoding wall-associated kinase is involved in wheat resistance to leaf rust similar to adult plant resistance
Source: PLoS One. 2020 Jan 13;15(1):e0227713. doi: 10.1371/journal.pone.0227713 (PMC6957155; doi:10.1371/journal.pone.0227713)
Supplement: S4 Fig — The kinase active site is highlighted in red. The blue hash sign indicates the arginine and non-arginine residues adjacent to the active site aspartic acid, RD and non RD respectively. (PDF) [file pone.0227713.s004.pdf]

```
AtWAK1                            YAESRILGQGGQGTVYKGILPDN-SIVAIKKARLGDSSQVEQFINEVLVLSQINHRNVVK    59
AtWAK2                            YHESRILGQGGQGTVYKGILPDN-SIVAIKKARLGNRSQVEQFINEVLVLSQINHRNVVK    59
TRIDC5BG010470.1                  LKNKKILGNGRFGTVSMGHTED-KQVVAVKEPINRK-STNDQFVNEIIIQSRVIHKNIVK    58
Tur_scaffold9692|24984-44913      LKNKKLLGNGHFGTVSMGHTED-KQVVAVKEPINRK-SPNDQFVNEIIIQSRVIHKNIVK    58
TraesCS5A02G052900.1              LKNKKLLGNGHFGTVSMGHTED-KQVVAVKEPINRK-SPNDQFVNEIIIQSRVIHKNIVK    58
TRIDC5AG008110.2                  LKNKKLLGNGHFGTVSMGHTED-KQVVAVKEPINRK-SPNDQFVNEIIIQSRVIHKNIVK    58
TRIDC5BG010460.1                  ----------------------------------------------------------LK    2
TraesCS5B02G063600.1              QKSSNIIGSGGFGKVYKGCIGDNNELVAVKEPINVNSANKGQFANEIIIQSPVIHRNIVK    60
TRIDC5BG010450.2                  QKSSNIIGSGGFGKVYKGCIGDNNELVAVKEPINVNSANKGQFANEIIIRSRVIHRNIVK    60
BRADI_2g03850v3                   LKASNVIGKGGFGMVYKGIIGDSSQLVAVKRPINVNLADQEQFANEIIIQSRVIHRNIVN    60
OsaBGIOSGA032260                  LKNANVIGKGGFGEVYKGHIGNNNQLVAVKKPINVNLAKKDQFANEVIIQSRVIHKNIVK    60
                                                                                            ::


AtWAK1                            LLGCCLETEVPLLVYEFITNGTLFDHLHGSMIDSSLTWEHRLKIAIEVAGTLAYLHSSAS    119
AtWAK2                            VLGCCLETEVPLLVYEFINSGTLFDHLHGSLYDSSLTWEHRLRIATEVAGSLAYLHSSAS    119
TRIDC5BG010470.1                  LIGCCLQFKVPTLIYEFVPKGSLDDILHGKNH-MPLKLGLRLQIAAESAEGLAYMHSKTT    117
Tur_scaffold9692|24984-44913      LIGCCLQFKVPTLIYEFVPNGSLDDILHGKNH-MPLKLGLRLQIAAESAEGLAYMHSKTT    117
TraesCS5A02G052900.1              LIGCCLQFKVPTLIYEFVPNGSLDDILHGKNH-MPLKLGLRLQIAAESAEGLAYMHSKTT    117
TRIDC5AG008110.2                  LIGCCLQFKVPTLIYEFVPNGSLDDILHGKNH-MPLKLGLRLQIAAESAEGLAYMHSKTT    117
TRIDC5BG010460.1                  LVGCCLQVEVPIFVYEFVPNGCLHDILHNGSR-MHLDMCKRLKIAAESAEGLAYMHSKTT    61
TraesCS5B02G063600.1              LVGCCLQVEVPILVYEFVPNGSLHDILHNGSR-MHLDMCKRLKIAAESAEGLAYMHSKTT    119
TRIDC5BG010450.2                  LVGCCLQVEVPILVYEFVPNGSLHDILHNGSR-MHLDMCKRLKIAAESAEGLAYMHSKTT    119
BRADI_2g03850v3                   LIGCCLEVDVPILVYEFVPKGSLHDILHGSHR-VPLDLNLRLQIAAESAEGLAYMHSKTA    119
OsaBGIOSGA032260                  LIGCCLEVDIPILVYEFVSKGSLEDVLHGSNR-LPLNLDQRLQIAAESAEGLAYMHSKTS    119
                                  ::****: .:* ::***: .* * * **.      *    **:** * *  ***:**.::
                                       #
AtWAK1                            IPIIHRDIKTANILLDVNLTAKVADFGASRLIPMDKE-ELETMVQGTLGYLDPEYYNTGL    178
AtWAK2                            IPIIHRDIKTANILLDKNLTAKVADFGASRLIPMDKE-QLTTIVQGTLGYLDPEYYNTGL    178
TRIDC5BG010470.1                  TTILHGDVKPANILLNEKFMPKISDFGISRLMVTD--MQHTGYIIFDRAYVDPVYRQTEQ    175
Tur_scaffold9692|24984-44913      TTILHGDVKPANILLNEKFMPKISDFGISRLMVTGMQMQHTGYIIFDKAYVDPVYRKTQQ    177
TraesCS5A02G052900.1              TTILHGDVKPANILLNEKFMPKISDFGISRLMVTGMQMQHTGCIIFDKAYVDPVYRKTQQ    177
TRIDC5AG008110.2                  TTILHGDVKPANILLNEKFMPKISDFGISRLMVTGMQMQHTGCIIFDKAYVDPVYRKTQQ    177
TRIDC5BG010460.1                  TTILHGDVKAANILLNDEFTPKISDFGISRLIVTD--MQHTGNVIGDMSYMDPVLLQTGL    119
TraesCS5B02G063600.1              TTILHGDVKPANILLNDEFTPKISDFGISRLIVTD--MQHTGNVIGDMSYMDPVLLQTGL    177
TRIDC5BG010450.2                  TTILHGDVKPANILLNDEFTPKISDFGISRLIVTD--MQHTGNVIGDMSYMDPVLLQTGL    177
BRADI_2g03850v3                   TTILHGDVKPANILLDDNFVPKISDFGISRLIATD--KQHTRNVIGDMSYMDPVYLQTGL    177
OsaBGIOSGA032260                  TTILHGDVKPANILLNDDLLPKISDFGISRLLAID--NDHTMSVIGDMSYMDPVYFQTGL    177
                                    *:* *:* *****: .:  *::*** ***:  .   :    :    .*:**   :*


AtWAK1                            LNEKSDVYSFGVVLMELLSGQKALCFKRPQSSKHLVSYFATATKENRLDEIIGGEVMNED    238
AtWAK2                            LNEKSDVYSFGVVLMELLSGQKALCFERPHCPKNLVSCFASATKNNRFHEIIDGQVMNED    238
TRIDC5BG010470.1                  LTTKSDIY-FGVVLLELITRKKASHSDNN-L---LGNFLDTYTKDKSVTELLDKELRED-    229
Tur_scaffold9692|24984-44913      LTTKSDIYSFGVVLLELITRKKASHSDNN-L---LGNFLDTYTKDKSVTELLDKELGED-    232
TraesCS5A02G052900.1              LTTKSDIYSFGVVLLELITRKKASHSDNN-L---LGNFLDTYTKDKSVTELLDKELGED-    232
TRIDC5AG008110.2                  LTTKSDIYSFGVVLLELITRKKASHSDNN-L---LGNFLDTYTKDKSVTELLDKELGED-    232
TRIDC5BG010460.1                  LTKKSDVYSFGVVLLELITRKKASHSDKNSL---LRNFLDAYTKDKSVIELVDKELAEV-    175
TraesCS5B02G063600.1              LTKKSDVYSFGVVLLELITRKKASHSDKNSL---LRNFLDAYTKDKSVIELVDKELAEV-    233
TRIDC5BG010450.2                  LTKKSDVYSFGVVLLELITRKKASHSDNNSL---LRNFLDAYTKDKSVIELVDKELAEV-    233
BRADI_2g03850v3                   LTNKSDVYSFGVVLLELITRKKASHSDNNSL---LMNFLDAYKNNKSVIELLDKELEVVQ    234
OsaBGIOSGA032260                  LTDKSDVYSFGVVLLELITRKKASHSDNNSL---LRNFLDAYTSGKTVTEFVDEEIAAAN    234
                                  *. ***:* *****:**:: :**   ..      * . : : .. : . *::. ::


AtWAK1                            NLKEIQEAARIAAECTRLMGEERPRMKEVAAKLEALRVEKTKHKWS       284
AtWAK2                            NQREIQEAARIAAECTRLMGEERPRMKEVAAELEAL----------       274
TRIDC5BG010470.1                  DQEILGHLVGMIMQCINLDVNQRPEMTDVAERLHDMV---------       266
Tur_scaffold9692|24984-44913      DQEILGHLIGMIMQCINLDVNQRPEMTDVAERLHDMV---------       269
TraesCS5A02G052900.1              DQEIFGHLIGMIMQCINLDVNQRPEMTDVAERLHDMV---------       269
TRIDC5AG008110.2                  DQEILGHLIGMIMQCINLDVNQRPEMTDVAERLHDMV---------       269
TRIDC5BG010460.1                  DREILDNLGEMIMQCLNLDVNQRPKMTDVAERLRDMV---------       212
TraesCS5B02G063600.1              DREILDNLGEMIMQCLNLDVNQRPEMTDVAERLRDMV---------       270
TRIDC5BG010450.2                  DREILDNLGEMIMQCLNLDVNERPEMTDVAERLRDMV---------       270
BRADI_2g03850v3                   DLELLDGLVGLIKQCLNLDVDQRPEMNDLVEQLRYM----------       270
OsaBGIOSGA032260                  DHELLVNLAGMIAQCLNLEVDQRPEMTDIAERLRYM----------       270
```

S4 Fig. Clustal Omega alignment of the closest WAK proteins. The kinase active site is highlighted in red. The blue hash sign indicates the arginine and non-arginine residues adjacent to the active site aspartic acid, RD and non RD respectively.
