# Supplementary material for: TaWAK6 encoding wall-associated kinase is involved in wheat resistance to leaf rust similar to adult plant resistance
Source: PLoS One. 2020 Jan 13;15(1):e0227713. doi: 10.1371/journal.pone.0227713 (PMC6957155; doi:10.1371/journal.pone.0227713)
Supplement: S1 Table — (PDF) [file pone.0227713.s009.pdf]

S1 Table. Primers and reaction conditions for PCR.

Primer symbol	Sequence	Conditions of PCR	Amplikon size (bp)
KR_5_Gf<br>KR_kl2Re	GTGAGATCACTGCATATCGAAAAG<br>CAAAACAATTTATTCGGGCAAACA	95ºC 3 min, 45x (95ºC 30 s, 60ºC 30 s, 72ºC 5 min),<br>72ºC 15 min	2355
q18S_Fw<br>q18S_Re	GTGACGGGTGACGGAGAATT<br>GACACTAATGCGCCCGGTAT	95ºC 15 min, 45x(95ºC 15 s, 58°C 20 s, 72ºC 25 s), 72ºC 1 min<br>72ºC – 95ºC (rising 1º) 5 s	150
R_I_77ant<br>KinR_5nR	GTTGGCGGACCTACATTGGAAAAGCTTA<br>CAAAACCACCACTTCCAATAATGTTGC	95ºC 15 min, 45x(95ºC 15 s, 58°C 20 s, 72ºC 25 s), 72ºC 1 min<br>72ºC – 95ºC (rising 1º) 5 s	105
bar7<br>bar8	TCTGCACCATCGTCAACCACTACATC<br>CAGAAACCCACGTCATGCCAGTTC	95ºC 12 min, 36x (95ºC 30 s, 60ºC 30 s, 72ºC 1 min), 72ºC 10 min	430
