# Supplementary material for: TaWAK6 encoding wall-associated kinase is involved in wheat resistance to leaf rust similar to adult plant resistance
Source: PLoS One. 2020 Jan 13;15(1):e0227713. doi: 10.1371/journal.pone.0227713 (PMC6957155; doi:10.1371/journal.pone.0227713)
Supplement: S2 Table — (PDF) [file pone.0227713.s010.pdf]

S2 Table. The list of *TaWAK* and *TaWAK-like* genes.

Gene	Accession number (NCBI)	Reference	Gene ID (Ensemble Plants)	Wheat subgenome	Query cover (NCBI)	E value (NCBI)	Ident (NCBI)	Numer of exons
TaWAK6	KR815340		TraesCS5B02G063600.1	5B	100%	0.0	99%	3
TaWAK6 homeoparalog 1			TraesCS5B02G063500.1 Nontranslating CDS	5B	88%	0.0	99%	3
TaWAK6 homeoparalog 2			TraesCS5B02G063700.1 Nontranslating CDS	5B	99%	0.0	82%	3
TaWAK6 homeoortolog			TraesCS5A02G052900.1	5A	99%	0.0	85%	3
TaWAK1	DQ013356	[24]	TraesCS2B02G464000.1	2B	100%	0.0	99%	3
TaWAK2	DQ013357	[24]	TraesCS3D02G518600.1 Nontranslating CDS	3D	100%	0.0	98%	3
			TraesCS2B02G231700.1	2B	97%	0.0	99%	3
TaWAK3	DQ013359	[24]	TraesCS7D02G085800.1	7D	94%	0.0	99%	3
TaWAK4	DQ013358	[24]	TraesCS3D02G518600.1 Nontranslating CDS	3D	100%	0.0	99%	3
			TraesCS2B02G231700.1	2B	99%	0.0	98%	3
TaWAK5	KF710462	[21]	TraesCSU02G171400.1	Un	90%	0.0	99%	3
			TraesCS4D02G042300.1	4D	**55%**	0.0	**92%**	3
TaWAK/Snn1	KP091701	[22]	TraesCS1B02G004100.1	1B	100%	0.0	100%	3
W5G2U8_WHEAT		[20]	only protein sequence in BLASTP TraesCS5D02G043400.1	5D				
TaWAKL1	DQ013355	[24]	TraesCS2A02G047600.1	2A	100%	0.0	99%	2
TaWAKL2	DQ177499	[24]	TraesCS2D02G046500.1	2D	98%	0.0	99%	2
WAKL2	KY485189	[19]	TraesCS3A02G049375.1	3A	100%	0.0	100%	3
WAKL3	KY485189	[19]	TraesCS3A02G049400.1	3A	100%	0.0	100%	3
TaWAKL4/Stb6	KY485188	[19]	TraesCS3A02G049500.2	3A	100%	0.0	100%	4
